# Genome-wide transcriptional profiling and functional analysis of long noncoding RNAs and mRNAs in chicken macrophages associated with the infection of avian pathogenic *E. coli*

**DOI:** 10.1186/s12917-024-03890-7

**Published:** 2024-02-07

**Authors:** Hongyan Sun, Xinqi Cao, Sumayya, Yuyi Ma, Huan Li, Wei Han, Lujiang Qu

**Affiliations:** 1 College of Animal Science and Technology, Yangzhou University, Yangzhou, 225009 China; 2 School of Biological and Chemical Engineering, Yangzhou Polytechnic College, Yangzhou, 225009 China; 3 The Poultry Research Institute of Chinese Academy of Agricultural Sciences, Yangzhou, 225009 China; 4 College of Animal Science and Technology, China Agricultural University, Beijing, 100091 China

**Keywords:** Avian pathogenic *E. coli*, lncRNA-mRNA, Immune and inflammatory response, lncRNA *TCONS_00007391*, *CD86*, RNAseq

## Abstract

**Background:**

Avian pathogenic *E. coli* (APEC) can cause localized or systemic infections, collectively known as avian colibacillosis, resulting in huge economic losses to poultry industry globally per year. In addition, increasing evidence indicates that long non-coding RNAs (lncRNAs) play a critical role in regulating host inflammation in response to bacterial infection. However, the role of lncRNAs in the host response to APEC infection remains unclear.

**Results:**

Here, we found 816 differentially expressed (DE) lncRNAs and 1,798 DE mRNAs in APEC infected chicken macrophages by RNAseq. The identified DE lncRNA-mRNAs were involved in Toll like receptor signaling pathway, VEGF signaling pathway, fatty acid metabolism, phosphatidylinositol signaling system, and other types of O-glycan biosynthesis. Furthermore, we found the novel lncRNA *TCONS_00007391* as an important immune regulator in APEC infection was able to regulate the inflammatory response by directly targeting *CD86*.

**Conclusion:**

These findings provided a better understanding of host response to APEC infection and also offered the potential drug targets for therapy development against APEC infection.

**Supplementary Information:**

The online version contains supplementary material available at 10.1186/s12917-024-03890-7.

## Background

Avian pathogenic *E. coli* (APEC), a causative agent of colibacillosis, can cause high mortality and significant economic losses in poultry industry worldwide. In general, chickens at 4–6 weeks are more susceptible to APEC, resulting in diarrhea, enteritis, varying degrees of septicemia, airsacculitis, meningitis, perihepatitis, swollen head syndrome, and pericarditis [1–3]. Moreover, APEC can also infect ducks, geese, pigeon, turkey, and game birds with similar clinical symptom of colibacillosis [4–6]. The poultry product contaminated by APEC could pose a global threat to human food safety. Currently, although antibiotics have been an effective method to control colibacillosis [7], the excessive and misuse of antibiotics resulted in multiple antibiotic-resistant strains, becoming a significant problematic in the poultry industry [8, 9]. Effective vaccines are the good methods to control colibacillosis. However, vaccination failure could often occur due to the different APEC strains or serotypes. Therefore, it is urgent to explore the host genetic immunity mechanism in order to prevent and control APEC infection.

Recently, accumulating evidence showed that lncRNAs, a group of non-coding RNA molecules of more than 200 nucleotides in length, can participate in a variety of biological processes by regulating gene expression at the epigenetic and post-transcriptional levels [10–12]. It was found that lncRNAs played an important role in cell polarization, inflammatory response, and disease process [13–16]. For example, Ahmad et al. demonstrated that the lncRNA *MALAT1*/microRNA-30b axis can regulate macrophage polarization and function [17]. Moreover, Ma et al. found that lncRNA *XIST* was able to regulate bovine mammary epithelial cell inflammatory response by modulating the NFκB/NLRP3 inflammasome pathway [18]. Currently, there are many published studies on lncRNAs associated with various virus infection in chicken. However, there is a lack of research on lncRNA in chicken during bacterial infections, particularly APEC infection.

Macrophages, a type of immune cell, are widely distributed in the blood and tissues and play an important role in body's immune defense, immune homeostasis, and immune surveillance. In the present study, to identify the lncRNAs/mRNAs expression profiles and reveal their important regulatory functions during APEC infection, we performed the high-throughput sequencing analysis to investigate the differentially expressed (DE) lncRNAs and mRNAs in chicken HD11 macrophages infected with or without APEC. Specifically, we determined the role of lncRNA *TCONS_00007391* and its target gene *CD86* upon APEC infection. We aimed to identify the important interactions between host lncRNAs-mRNAs and APEC infection, which may contribute to a better understanding of host immune response and provide potential biomarkers and therapeutic targets for APEC infection.

## Results

### Effect of APEC infection on inflammatory response of chicken macrophages

**Fig. 1:**
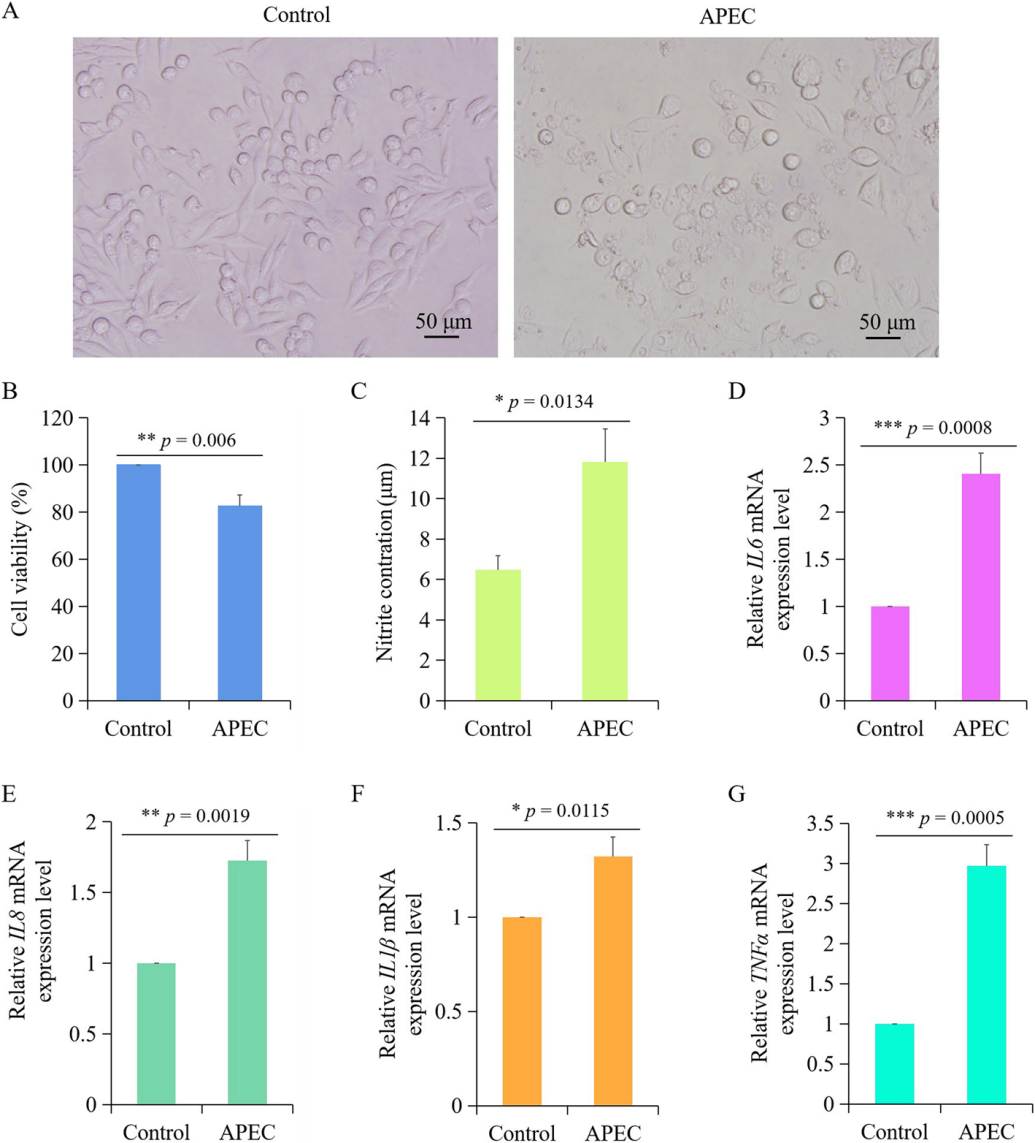
Effect of avian pathogenic *E. coli* (APEC) infection on immune response of chicken macrophages. **A** The morphology of chicken macrophages with or without APEC infection. **B** The cell viability of chicken macrophages before and after APEC infection. **C** The nitric oxide (NO) production of chicken macrophages before and after APEC infection. D-G. The mRNA expression level of *IL6* (**D**), *IL8* (**E**), *IL1β* (**F**), and *TNFα* (**G**) in chicken macrophages before and after APEC infection. Data are shown as mean ± SD; n = 4 independent experiments; * p < 0.05; ** *p* < 0.01; *** *p* < 0.001; NS, not significant

Morphological changes of chicken HD11 macrophages were observed with or without APEC infection. As shown in Fig. 1A, cytopathic effects were observed in HD11 macrophages infected with APEC at 10^8^ cfu/mL for 24 h in comparison to the Control group. In Control group, the wall-adherent macrophages were spindle-shaped or polygonal, while non-adherent ones were rounded (Fig. 1A). However, cells were swelled, deformed or destroyed after APEC infection (Fig. 1A). Moreover, cell survival rate was significantly decreased in APEC infected chicken macrophages measured by an CCK8 assay (Fig. 1B), which was consistent with the results of cell morphology. Additionally, NO production was significantly increased in APEC infection group (Fig. 1C). In terms of inflammatory factors, the expression level of *IL6* exhibited significantly higher levels in chicken macrophages infected with APEC (Fig. 1D). Similarly, the expression of *IL8*, *IL1β*, and *TNFα* were also significantly increased in APEC infection group (Fig. 1E-G). These results indicated that APEC infection could significantly induce the inflammatory response in chicken macrophages.

### Analysis of lncRNAs and mRNAs profile during APEC infection

**Fig. 2:**
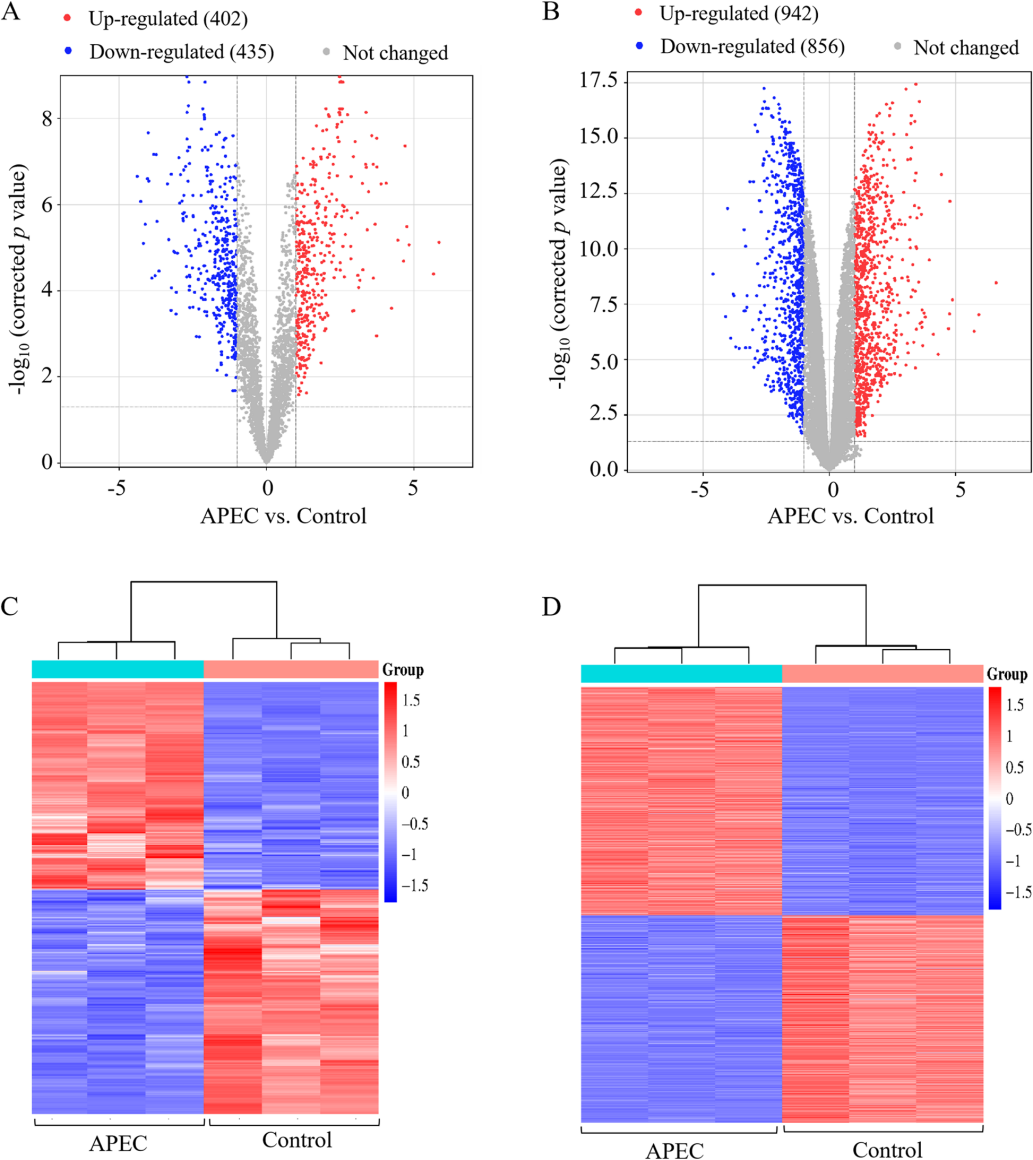
The differentially expressed (DE) lncRNAs and mRNAs in APEC infected macrophages (APEC) vs. non-infected macrophages (Control). **A-B** Volcano plot diagram of the DE lncRNAs (**A**) and mRNAs (**B**) between APEC infected macrophages and non-infected macrophages. **C-D** Hierarchical clustering of the DE lncRNAs (**C**) and mRNAs (**D**)

To identify the significantly differentially expressed (DE) lncRNAs and mRNAs in the comparison of APEC infected chicken macrophages (APEC) vs. non-infected chicken macrophages (Control), six cDNA libraries were constructed for whole transcriptome sequencing. In total, 816 DE lncRNAs (401 up-regulated and 415 down-regulated) and 1,798 mRNAs (946 up-regulated and 858 down-regulated) were identified, respectively, with a corrected* p* value < 0.05 and a |log_2_ (fold change)|> 1 as the cutoff values in APEC group compared to the Control group (Fig. 2A-B). Hierarchical clustering showed that the expression level of the identified DE lncRNAs and mRNAs in APEC group was significantly different from those in Control group. These results indicated that the altered expression of the DE lncRNAs and mRNAs was caused by APEC infection (Fig. 2C-D).

### Functional analysis of the identified DE genes

**Fig. 3:**
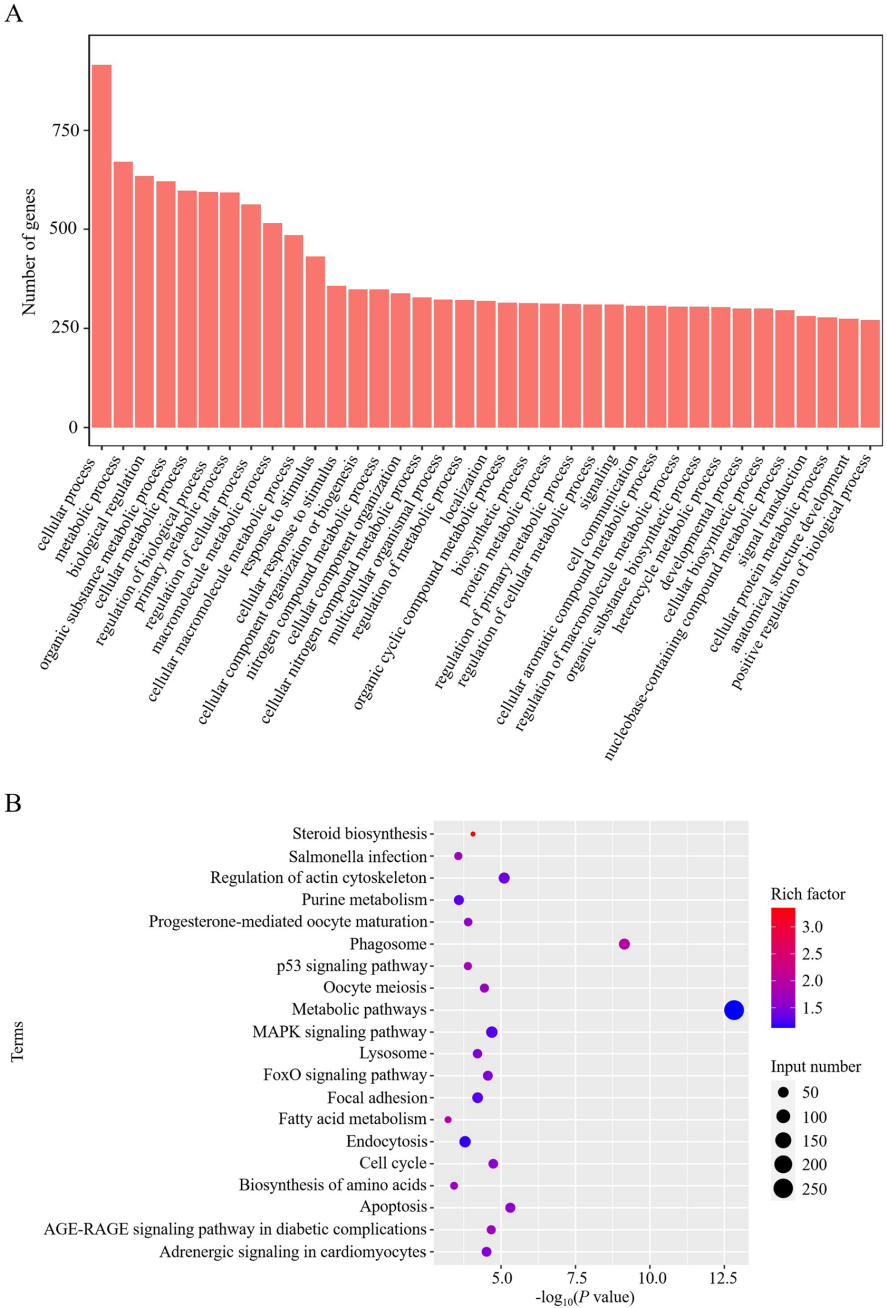
GO and KEGG pathway analyses of the differentially expressed (DE) genes in avian pathogenic *E. coli* infected macrophages (APEC) vs. non-infected macrophages (Control). A GO analysis of the DE genes in APEC vs. Control. B KEGG analysis of the DE genes in APEC vs. Control

To better understand and predict the biological function and corresponding pathways of the identified significant DE genes in APEC vs. Control, GO enrichment and KEGG pathways were performed to explore the potential functions and regulatory networks. According to the GO annotation, the biological process included cellular process, biological regulation, response to stimulus, cell communication, signal transduction (Fig. 3A). KEGG analysis showed that a total of 65 pathways were identified, including the Phagosome, p53 signaling pathway, MAPK signaling pathway, Lysosome, Focal adhesion, Endocytosis, and Apoptosis etc. immune related pathways. The top 20 enriched pathways of the identified DE genes are shown in Fig. 3B. These results indicated that the DE genes might play a crucial role in chicken against APEC infection.

### Prediction the target genes of the identified DE lncRNAs

**Fig. 4:**
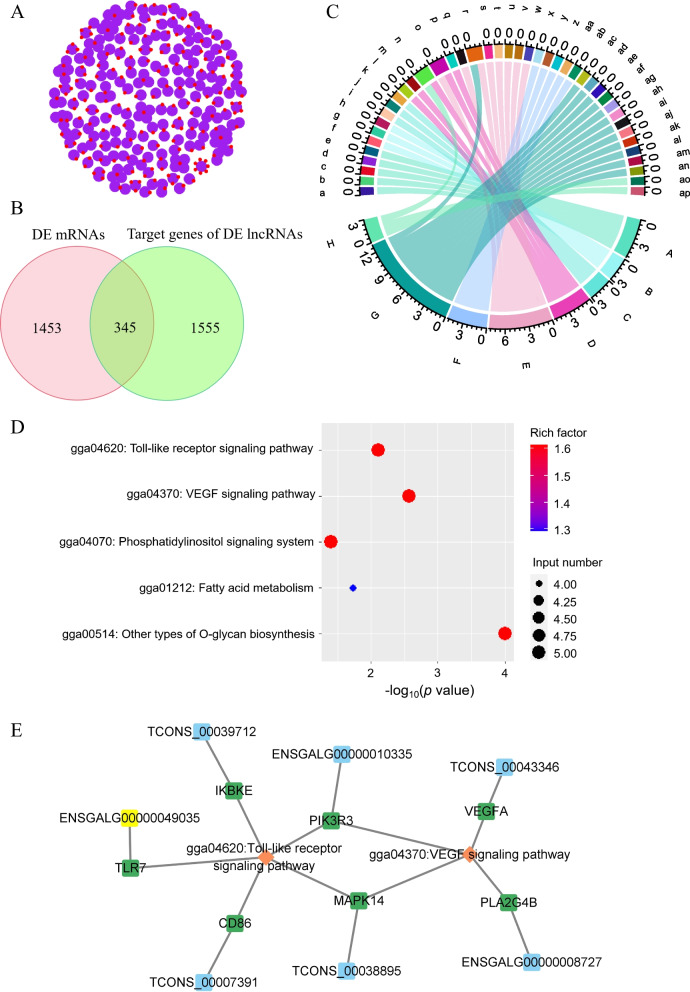
Functional analysis of the overlapping genes between the differentially expressed (DE) mRNAs and the potential target genes of DE lncRNAs in avian pathogenic *E. coli* infected macrophages (APEC) vs. non-infected macrophages (Control). A. The potential target genes of the identified DE lncRNAs. B. Venn diagram of the number of the overlapping genes between the DE mRNAs and the target genes of DE lncRNAs in APEC vs. Control. C. GO analysis of the overlapping genes between the DE mRNAs and the target genes of DE lncRNAs in APEC vs. Control. a, *P2RY8*; b, *GPR35L*; c, *GPR35*; d, *GPR65*; e, *LOC112532977*; f, *LFNG*; g, *GALNT11*; h, *MFNG*; i, *SLC12A4*; j, *ADD1*; k,* SLC12A9*; l, *JUP*; m, *PRKCB*; n,* BRCA1*; o, *RHOB*; p, *VEGFA*; q,*CD86*; r, *TNFSF15*; s, *LY86*; t, *TNFSF8*; u, *TLR7*; v, *CTSV*; w, *CD244*; x, *RASGEF1A*; y, *RASGEF1B*; z, *DOCK8*; aa, *LOC429518*; ab, *RND2*; ac, *CHRNA1*; ad, *PHLPP1*; ae, *LOC428967*; af, *LRRK1*; ag, *FAM13A*; ah, *AFDN*; ai, *RASSF5*; aj, *IL1RAPL2*; ak, *RHPN1*; al, *TCP11L1*; am, *SRGAP2*; an, *PLPP1*; ao, *BCL2A1*; ap, *IKBKE*; A, positive regulation of Rho protein signal transduction; B, regulation of Notch signaling pathway; C, cell volume homeostasis; D, positive regulation of angiogenesis; E, immune response; F, small GTPase mediated signal transduction; G, signal transduction; H, intrinsic apoptotic signaling pathway in response to DNA damage. D. KEGG analysis of the overlapping genes between the DE mRNAs and the target genes of DE lncRNAs in APEC vs. Control. E. Cytoscape visualizing of the overlapping DE genes and their corresponding lncRNAs that enriched in the immune related pathway

Functional roles of the identified DE lncRNAs were investigated by examining the cis-regulated and trans-regulated mRNAs. Results showed that a total of 1,900 target genes were predicted for 466 known lncRNAs and 203 novel lncRNAs with differential expression in the comparison of APEC vs. Control (Fig. 4A). In addition, a total of 345 overlapping mRNAs were identified between the DE lncRNA target genes and the DE genes (Fig. 4B). Then, GO and KEGG analyses of the 345 overlapping DE mRNAs were further used to annotate their functions. Results showed that the overlapping DE genes were significantly enriched in the biological processes, including regulation of Notch signaling pathway, cell volume homeostasis, immune response, positive regulation of angiogenesis, small GTPase mediated signal transduction, signal transduction, and intrinsic apoptotic signaling pathway in response to DNA damage (Fig. 4C). Furthermore, KEGG analysis showed that the overlapping DE genes were grouped into the functional categories of other types of O-glycan biosynthesis, VEGF signaling pathway, Toll-like receptor signaling pathway, fatty acid metabolism, and phosphatidylinositol signaling system (Fig. 4D). Meanwhile, the overlapping DE genes and their corresponding lncRNAs that enriched in the immune related pathways were visualized by using Cytoscape (Fig. 4E). The aforementioned results indicated that the induced lncRNAs-mRNAs regulated immunity, signal transduction, and apoptosis during APEC infection.

### RT-qPCR analysis of the lncRNA-mRNA pairs

**Fig. 5:**
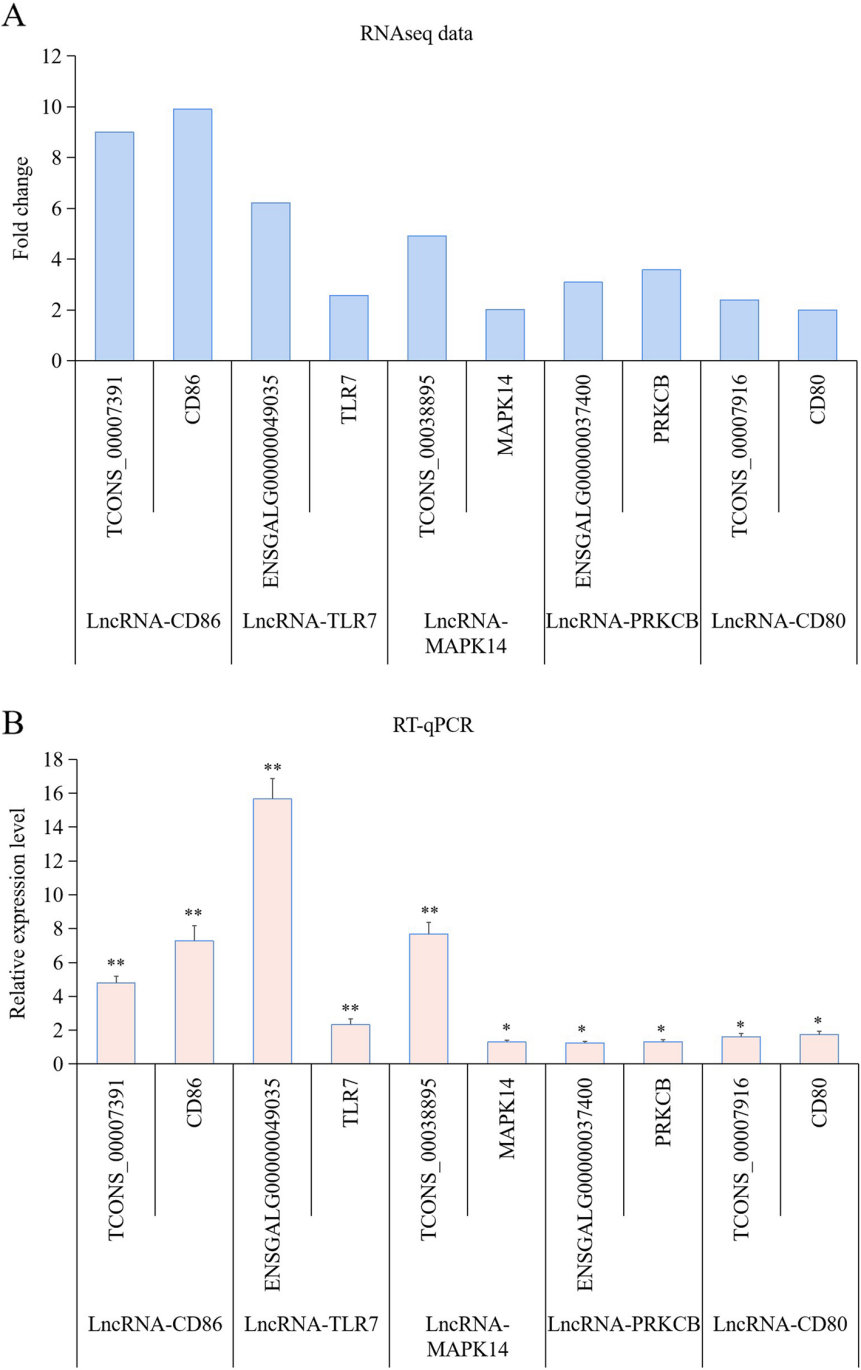
RT-qPCR was used to validate the RNAseq data. **A** The fold change of the differentially expressed (DE) lncRNA-mRNA pairs in RNAseq data. **B** The relative expression level of the differentially expressed (DE) lncRNA-mRNA pairs in RT-qPCR experiment

To validate the predicted lncRNA-mRNA pairs, we randomly selected five DE lncRNAs and their five target mRNAs (the DE genes) for RT-qPCR analysis. Results showed that the relative changes in the expression levels of lncRNAs and their target mRNAs (the DE genes) expression levels were consistent with those detected in RNAseq (Fig. 5). These results indicated that the RNAseq data, together with the predicted lncRNA − mRNA pairs, were reliable and accurate.

### LncRNA *TCONS_00007391* and *CD86* were up-regulated during APEC infection

**Fig. 6:**
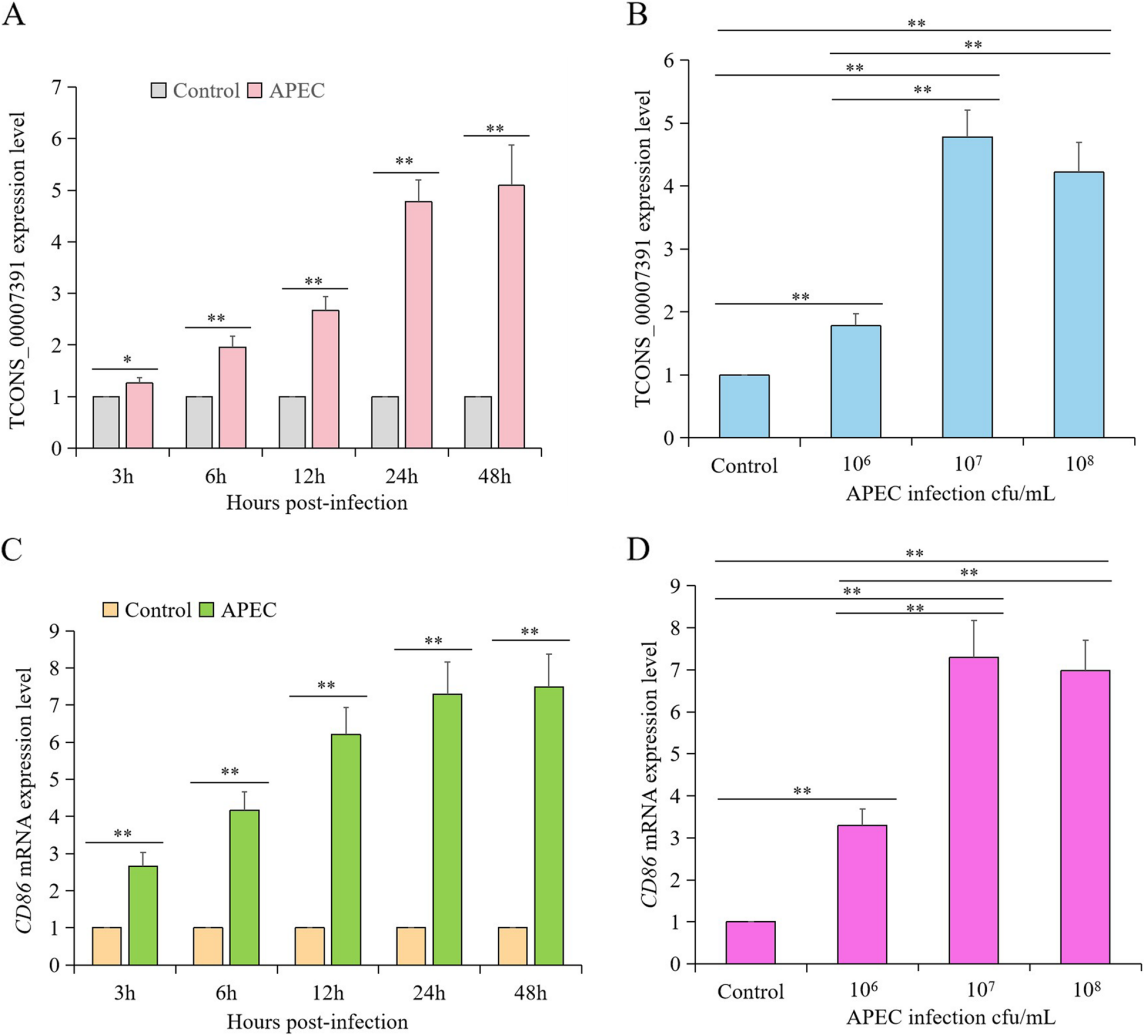
The correlation between lncRNA *TCONS_00007391* and *CD86* upon APEC infection. A and C. The expression level of lncRNA *TCONS_00007391* (**A**) and *CD86* (**C**) after chicken macrophages infected with APEC (1 × 10^7^ cfu/mL) for 3 h, 6 h, 12 h, 24 h, and 48 h by using RT-qPCR. **B** and **D** The expression level of lncRNA *TCONS_00007391* (**B**) and *CD86* (**D**) after chicken macrophages infected with APEC at different concentrations (0, 10^6^ cfu/mL, 10^7^ cfu/mL, and 10^8^ cfu/mL) for 24 h via RT-qPCR. data represent the mean ± SD of four independent experiments. * *p* < 0.05; ** *p* < 0.01

The pair of lncRNA TCONS_00007391 and *CD86* were selected for further study because they were predicted to be involved in Toll-like receptor signaling pathway based on the functional analysis of DE lncRNAs and mRNAs. We first investigated the correlation between lncRNA *TCONS_00007391* and *CD86* by examining their transcript levels in chicken macrophages infected with APEC at different time points. The expression of *TCONS_00007391* was significantly increased in an APEC dose- and infection time-dependent manner. At a concentration of 1 × 10^7^ cfu/mL APEC, the expression of *TCONS_00007391* was significantly increased at 12 h, and peaked at 24–48 h (Fig. 6A). After APEC (1 × 10^6^ cfu/mL) infection for 24 h, the *TCONS_00007391* expression level was significantly higher than that in Control group (Fig. 6B). The expression level of *TCONS_00007391* reached its peak with 1 × 10^7^ cfu/mL APEC infection, while it decreased with 1 × 10^8^ cfu/mL APEC infection (Fig. 6B). In addition, *CD86*, the target gene of *TCONS_00007391*, was also significantly up-regulated in an APEC dose-dependent (Fig. 6C) and infection time-dependent manner (Fig. 6D). These results indicated that a positive correlation existed between *TCONS_00007391* and *CD86*.

### Expression pattern of LncRNA *TCONS_00007391* and *CD86* in different tissues

**Fig. 7:**
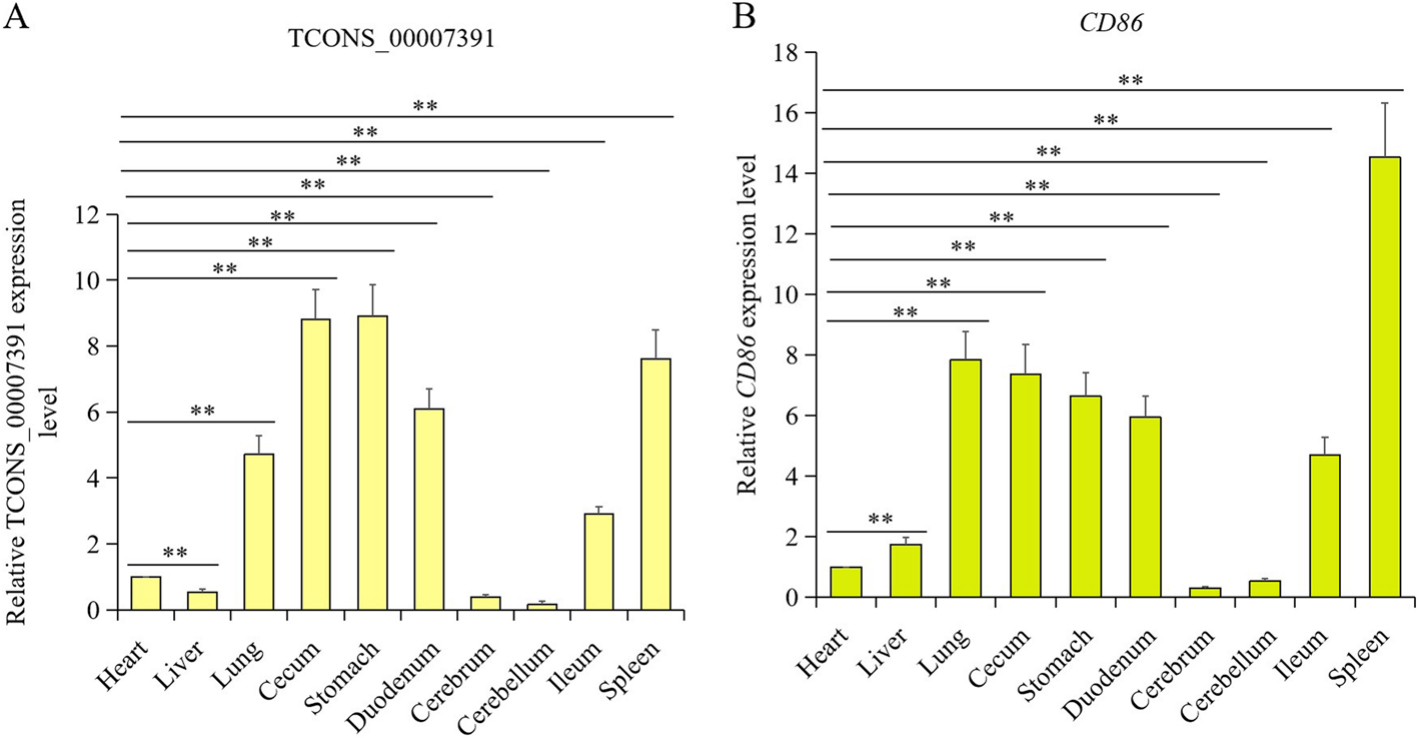
Relative expression pattern of lncRNA *TCONS_00007391* and *CD86* gene in chicken different tissues. A-B. The relative expression level of *TCONS_00007391* (**A**) and *CD86* (**B**) in heart, liver, lung, cecum, stomach, duodenum, cerebrum, cerebellum, ileum, and spleen were measured by using RT-qPCR. The result was normalized with *GAPDH* gene and relative to gene expression in the heart group. Data are shown as mean ± SD; n = 8; ** indicates *p* < 0.01

Total RNA was isolated from ten tissues (heart, liver, lung, cecum, stomach, duodenum, cerebrum, cerebellum, ileum, and spleen). The expression level of *TCONS_00007391* and *CD86* in different tissues was determined by RT-qPCR. As shown in Fig. 7A, the *TCONS_00007391* expression level was significantly higher in lung, cecum, stomach, duodenum, ileum, and spleen in comparison to heart (*p* < 0.01). Furthermore, it is worth noting that *CD86*, a lncRNA *TCONS_00007391* potential target mRNA, also had significant higher expression level in lung, cecum, stomach, duodenum, ileum, and spleen compared to heart (*p* < 0.01) (Fig. 7B). These results indicated that *TCONS_00007391* and *CD86* had the same expression pattern in chicken different tissues.

### Construction of overexpression and interference vector of lncRNA *TCONS_00007391*

**Fig. 8:**
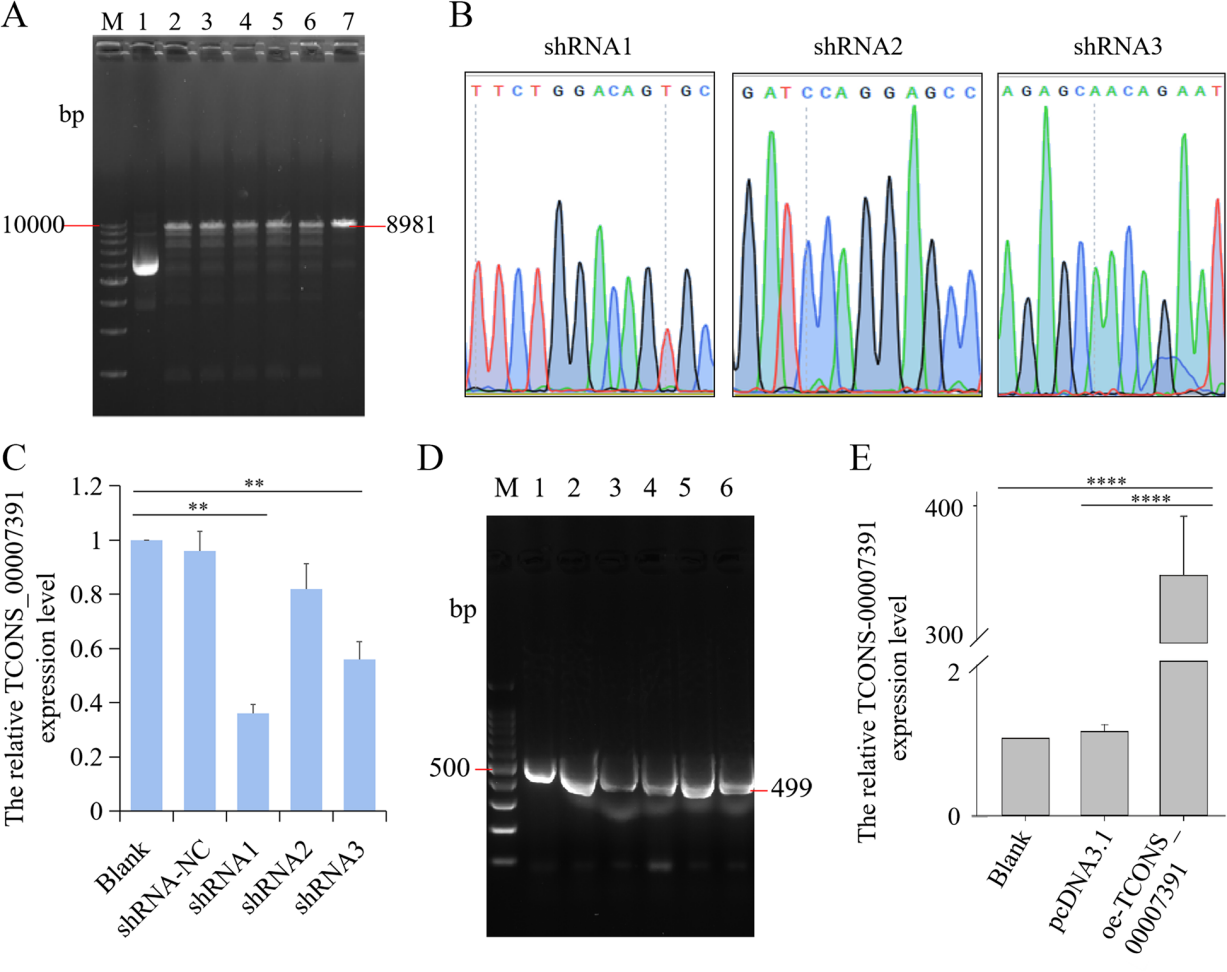
Construction and activity verification of *TCONS_00007391* RNA interference/overexpression vector. **A** Double enzyme digestion of the *TCONS_00007391* interference recombination plasmid. M, Marker; 1, empty vector; 2–3, shRNA1; 4–5, shRNA2; 6–7, shRNA3. **B** Sanger sequencing of the *TCONS_00007391* interference recombination plasmid. **C** Relative expression of *TCONS_00007391* in macrophages transfected with shRNA vectors for 48 h as measured by RT-qPCR. **D** Bacterial liquid PCR amplification of *TCONS_00007391*. M, Marker; 1–6, *TCONS_00007391*. **E** Relative expression of *TCONS_00007391* in macrophages transfected with overexpression vector for 48 h as measured by RT-qPCR

To identify the potential role of lncRNA *TCONS_00007391* in APEC infection, we designed and constructed the specific interference and overexpression vector of *TCONS_00007391*. As shown in Fig. 8A-B and supplementary file 1-2, the interference recombination plasmid was successfully constructed according to the results of double enzyme digestion electrophoresis gel and Sanger sequencing. At 48 h after transfection, the shRNA1 and shRNA3 of lncRNA *TCONS_00007391* can markedly decrease the expression level of *TCONS_00007391* compared with the blank group (*p* < 0.01) (Fig. 8C). The shRNA1 of *TCONS_00007391* was used for follow-up experiments due to its strongest interference activity. Meanwhile, bacterial liquid PCR amplification showed that the overexpression recombination plasmid of *TCONS_00007391* was also successfully obtained (Fig. 8D, supplementary file 3-4). The expression of *TCONS_00007391* was significantly increased after macrophages transfected with the overexpression plasmid in comparison to blank group (*p* < 0.0001) (Fig. 8E).

### Effect of lncRNA *TCONS_00007391* on APEC infected macrophages

**Fig. 9:**
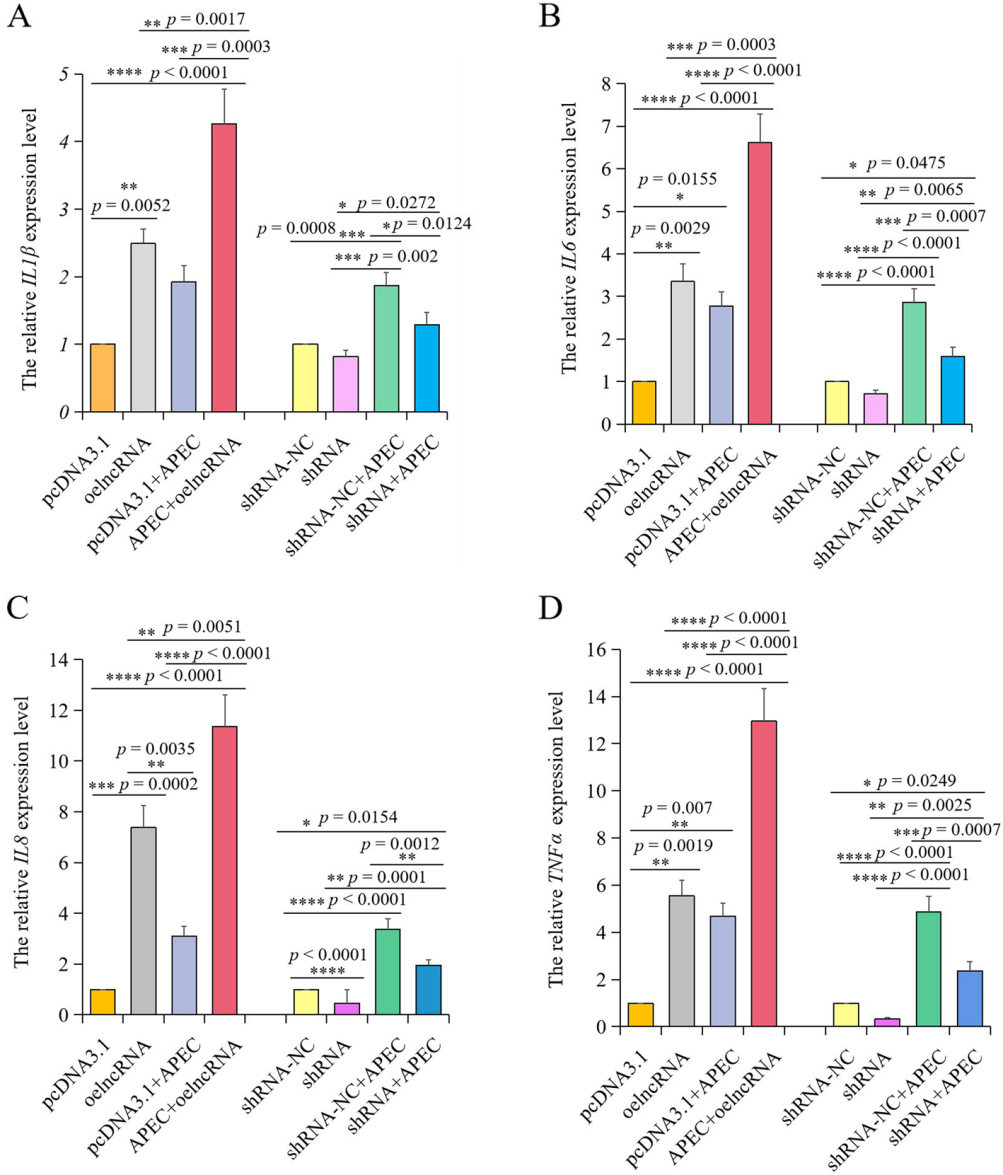
The effects of lncRNA *TCONS_00007391* on different cytokines in chicken macrophages with or without APEC infection. **A-D** The expression levels of four pro-inflammatory mediators, including *IL1β* (**A**), *IL6* (**B**), *IL8* (**C**), and *TNFα* (**D**) were analyzed using RT-qPCR after chicken macrophages were transfected with the overexpression/interference of TCONS_00007391 vector associated with or without APEC infection. Data are shown as mean ± SD; n = 4 independent experiments; * *p* < 0.05, ** *p* < 0.01, *** *p* < 0.001, **** *p* < 0.0001

**Fig. 10:**
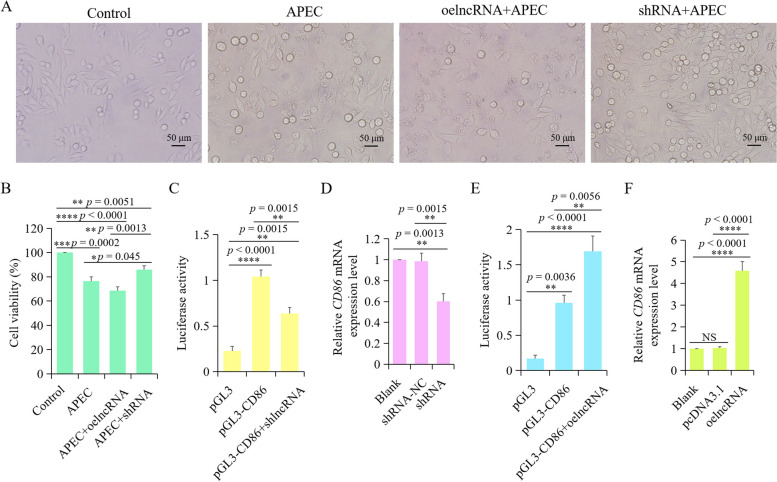
*TCONS_00007391* was able to regulate avian pathogenic *E. coli* (APEC) induced cell viability via targeting *CD86*. **A** The morphology of chicken macrophages in the groups of Control, APEC, ovexpression of *TCONS_00007391* + APEC, and knockdown of *TCONS_00007391* + APEC. **B** The cell viability of chicken macrophages in the groups of Control, APEC, ovexpression of *TCONS_00007391* + APEC, and knockdown of *TCONS_00007391* + APEC. Data are shown as mean ± SD; n = 4 independent experiments; * *p* < 0.05, ** *p* < 0.01, *** p < 0.001, **** *p* < 0.0001. **C** Luciferase reporter assay was used to determine the relationship between knockdown of *TCONS_00007391* and *CD86*. Firefly luciferase activities were normalized to Renilla luciferase activities. Data are shown as mean ± SD; n = 4 independent experiments; ** *p* < 0.01, **** *p* < 0.0001. **D** RT-qPCR analysis of *CD86* mRNA expression in macrophages transfected with the *TCONS_00007391* knockdown plasmid. Data are shown as mean ± SD; n = 4 independent experiments; ** *p* < 0.01, **** *p* < 0.0001. **E** Luciferase reporter assay was used to determine the relationship between overexpression of *TCONS_00007391* and *CD86*. Firefly luciferase activities were normalized to Renilla luciferase activities. Data are shown as mean ± SD; n = 4 independent experiments; ** *p* < 0.01, **** *p* < 0.0001. **F** RT-qPCR analysis of *CD86* mRNA expression in macrophages transfected with the *TCONS_00007391* overexpression plasmid. Data are shown as mean ± SD; n = 4 independent experiments; **** *p* < 0.0001

As shown in Fig. 9, lncRNA *TCONS_00007391* interference can significantly suppress the expression level of *TNFα*, *IL8*, *IL6*, and *IL1β* with or without APEC infection, while overexpression of *TCONS_00007391* can significantly increase the expression of those cytokines. After silencing/overexpressing lncRNA *TCONS_00007391*, both morphology and viability of chicken macrophages with or without APEC infection were examined to identify the function of lncRNA by using microscope and CCK8 assay, respectively. Results showed that cytopathic effects occurred in chicken macrophages after APEC infection in comparison to the Control group. Knockdown of lncRNA *TCONS_00007391* can significantly attenuate the APEC induced cytopathy, whereas overexpression of *TCONS_00007391* was able to exacerbate the APEC induced cellular injury (Fig. 10A). Furthermore, it was found that overexpression of *TCONS_00007391* can significantly reduce the APEC induced cell survival rate in comparison to APEC and Control group (Fig. 10B). However, knockdown of lncRNA *TCONS_00007391* was able to rescue the APEC induced cell survival rate compared to the APEC infection group (Fig. 10B). Therefore, these results suggested that knockdown lncRNA *TCONS_00007391* could alleviate the inflammatory response and increase cell viability during APEC infection.

### *CD86* was the target of lncRNA *TCONS_00007391*

According to the results of RNAplex, *CD86*, which is differentially expressed upon APEC infection, was predicted to be the potential target of lncRNA *TCONS_00007391*. Dual luciferase reporter gene system was first used to identify the interaction between lncRNA *TCONS_00007391* and *CD86*. It was found that luciferase activity was significantly decreased in the macrophages transfected with *TCONS_00007391* knockdown plasmid and pGL3-CD86, whereas it was significantly increased in the macrophages transfected with *TCONS_00007391* overexpression plasmid and pGL3-CD86 (Fig. 10C and E). Moreover, results of RT-qPCR showed that the *CD86* mRNA expression level was significantly declined in the knockdown of lncRNA *TCONS_00007391* compared with the negative control (Fig. 10D), while markedly increased in the overexpression of *TCONS_00007391* group (Fig. 10F). The aforementioned results indicated that lncRNA *TCONS_00007391* was able to regulate the expression of *CD86*.

## Discussion

APEC, a group of gram-negative bacteria, is responsible for causing avian diseases, which can be acute or chronic. These diseases result in significant economic losses in poultry industry [19–21]. However, the underlying molecular mechanisms of host immune response against APEC infection are still poorly understood. Recently, accumulated studies have demonstrated that the lncRNAs are widely involved in various physiological and pathological processes, including bacterial infection [22–24]. However, little is known for the role of lncRNAs during APEC infection. Macrophages are key cells that initiate inflammation. They can participate in the inflammatory response by activating immune system and releasing a series of inflammatory mediators, such as cytokines. Therefore, in this study, transcriptome analysis of lncRNAs and mRNAs was investigated in APEC infected chicken macrophages, among which the function of novel lncRNA, *TCONS_00007391* was specially validated. This study was the first to investigate the complex regulation of lncRNAs in APEC infected chicken macrophages.

In current study, the DE lncRNAs and DE genes identified by the transcriptome analysis were involved in a high consistency of pathways, resulting in relatively more reliable target relationships between lncRNAs and mRNAs. Both the up-regulated lncRNAs and mRNAs are mainly involved in immune pathway related to Toll-like receptor signaling pathway, indicating the potential synergistic effect of lncRNA and mRNA. It has been demonstrated that Toll-like receptor (TLR) signaling pathway, which is essential for the innate immune system, is involved in recognition of the bacteria and induction of the reactive oxygen species (ROS), type I IFN, NFκB activation of proinflammatory cytokines [25–27].

The pair of *TCONS_00038895*-*MAPK14* was detected in the aforementioned pathway (TLR signaling pathway). MAPK14 is particularly important in the regulation of inflammation and cell death [28–30]. MAPK14 has shown to regulate the lipopolysaccharide (LPS) induced lung cell injury [31]. In present study, the fold change of lncRNA *TCONS_00038895* and *MAPK14* was 4.92 and 2.02, respectively. The pair of *TCONS_00007391* and *CD86* was also involved in the Toll-like receptor signaling pathway with a fold change of 8.99 and 9.92, respectively. Moreover, the expression pattern of *TCONS_00007391* and *CD86* was similar in chicken different tissues, indicating their highly positive correlation. Researchers have demonstrated that lncRNAs had function to cooperate with neighboring genes to perform cis regulatory function [11, 32, 33]. Furthermore, Dimitrova et al. found that *LincRNA-p21* was able to regulate *p21* in cis form to modulate the activation and chromatin state of hundreds of downstream genes [34]. In present study, we also found that the novel lncRNA *TCONS_00007391* was capable of cis-regulating of the expression level of *CD86*.

CD86, a member of the immunoglobulin superfamily, is constitutively expressed on the immune cells, including dendritic cells, macrophages, B cells and other antigen-presenting cells [35–39].CD86 can bind to CD28 to provide costimulatory signals necessary for T cell activation and survival [40, 41]. CD86 can also bind to the CTLA-4 receptor on T cells to inhibit T cell activation [42–44]. In present study, *CD86* had a significantly higher expression level during APEC infection, indicating both the innate and acquired immunity were highly activated during APEC infection.

Since lncRNAs could regulate the immune response against bacterial infection [45–47], we also validated the function of novel lncRNA *TCONS_00007391*, a regulator of *CD86* identified in this study, by gene silencing and overexpression. Results showed that knockdown of *TCONS_00007391* can rescue the APEC induced cellular injury, while overexpression of *TCONS_00007391* can exacerbate the APEC induced cellular injury. Altogether, the aforementioned findings provide new directions for better understanding of host response to APEC infection and offer new insights for the preventing and treating of APEC infection.

## Conclusion

In summary, we investigate the transcriptional profiles of lncRNAs and mRNAs in chicken macrophages upon APEC infection by RNAseq. A total of 816 DE lncRNAs and 1,798 DE mRNAs were identified to be associated with APEC-host interactions. Moreover, the overlapping genes between DE lncRNAs and DE mRNAs were enriched in Toll like receptor signaling pathway and VEGF signaling pathway, suggesting that the lncRNA-mRNA pairs were involved in chicken immune related pathways against APEC infection. Furthermore, the identified novel lncRNA *TCONS_00007391* was able to regulate the APEC induced inflammatory response by directing targeting *CD86*. The present findings not only provide a new perspective for chicken in response to APEC infection, but also offer the potential drug targets for therapy development against APEC infection.

## Materials and methods

### APEC O78

The bacteria strain of APEC O78 was from Chinese Veterinary Culture Collection Center (CVCC, Beijing, China). LB agar plate was used to grow APEC O78. Then, a single colony was picked to LB medium for continuously culturing at 37 °C overnight. Before APEC infection, the bacteria culture medium was centrifugated at 5000 × g for 15 min to obtain the APEC O78 pellets. Then, the pellets were washed three times by using PBS. Bacteria were counted based on spectral readings at 600 nm, and the inoculum was adjusted to the desired bacterial concentration in PBS. Counts were confirmed by plating serial dilutions of the inoculum on MacConkey agar overnight. The gradient test of a previous infection experiment indicated that 0.1 mL of the 1 × 10^8^ cfu/mL concentration of APEC O78 was the most suitable dosage to induce cellular immune response [48].

### Cell culture

The HD11 macrophages were grown in RPMI1640 (Gibco, Carlsbad, CA, USA) supplemented with 10% fetal bovine serum (FBS, Gibco, Carlsbad, CA, USA) in a humidified incubator with 5% CO_2_ at 37 °C. Macrophages were passaged before 80–90% confluence.

### APEC infection

Macrophages (1 × 10^5^ cells/well) were seeded in 48-well plates and divided into Control and APEC group. The Control group indicated that chicken macrophages did not infect with APEC. For APEC group, cells were challenged with 0.1 mL APEC O78 (1 × 10^8^ cfu/mL) for 24 h. The cells were observed under an optical microscope (Olympus, Japan). Then, supernatant was discarded and cells were washed with PBS for two times. Cells were digested by trypsin for 2 min and culture medium was used to stop the digestion. Cells were collected after centrifugation at 626 × g for 6 min. The harvested cells were used for subsequent experiment.

### Detection of inflammatory factors expression levels in macrophages infected with APEC

Macrophages from Control and APEC group were used to isolate the total RNA by Trizol reagent (Invitrogen, Carlsbad, CA, USA). The quality and concentration of the extracted RNA was detected by Nanodrop ND-1000 spectrophotometer (Thermo Fisher ScientificInc., MA, USA). Then, the RNA was reverse transcribed into cDNA using a Reverse Transcription Kit (Takara, Dalian, China). cDNA was synthetized using the One Step SYBR® PrimeScript® PLUS RTRNA PCR Kit (Takara, Dalian, China). RT-qPCR was used to identify the expression level of inflammatory factors (*IL1β*, *IL8*, *IL6*, and *TNFα*). Primer sequences of the inflammatory factors were displayed in Table S1.

### Total RNA Extraction, cDNA library, and RNA sequencing

RNA isolation kit (QIAGEN, Hilden, Germany) was used to extract the total RNA from chicken macrophages with or without APEC infection. Then, agarose gel electrophoresis and a Nanodrop™ OneCspectrophotometer (Thermo Fisher Scientific Inc., MA, USA) were used to determine the quality of total RNA. RNA integrity number (RIN) was also detected by Qseq (Qseq100, Guangding, Taiwan). Ribo-off rRNA depletion kit (Illumina, San Diego, CA, USA) was used to construct the stranded RNA sequencing libraries with 2 μg of the qualified RNA. The quality of the libraries was determined by Qubit 3.0 with Qubit™ RNA Broad Range Assay kit (Life Technologies, Carlsbad, CA, USA). Then, the libraries with 200–500 bps were sequenced on NovaSeq 6000 sequencer (Illumina, San Diego, CA, USA) with PE150 sequencing platform.

### Analysis of RNA sequencing data

Trimmomatic (version 0.36) was used to filter the low-quality reads and adaptor sequences. The duplicated reads introduced in PCR amplification or sequencing were also removed. After that, STRA software (version 2.5.3a) was used to map the deduplicated clean reads to chicken reference genome (*gallus gallus 6*: https://asia.ensembl.org/info/data/ftp/index.html). New transcripts (lncRNAs) were identified by using Stringtie (version 1.3.2). The criteria for identifying new lncRNAs include transcripts that are longer than 200 bp and have more than 2 exons and 3 reads. Then, the coding potential of new transcripts was predicted by CPC2 (version 2.0), CPAT (version 1.2.4), CNCI (version 2), and Pfam (version 27.0). FeatureCounts (Subread-1.5.1, Bioconductor) was used to determine the expression of novel and known lncRNAs. Finally, reads per kilobase of exon model per million mapped reads (RPKM) was used for normalization.

EdgeR package (version 3.12.1) [49] was used to identify the differentially expressed (DE) lncRNAs and mRNAs between APEC and Control group. The threshold of the statistical significance of DE lncRNAs or DE mRNAs was the adjusted *p* value less than 0.05 and |log_2_(fold-change)| more than 1. RIsearch (version 2.0) [50] was used to predict the DE lnRNAs anti-sense targets with the criteria of the minimum binding free energy less than –50. Meanwhile, the potential cis targets were investigated in 100 kb upstream and downstream of the DE lncRNAs. WGCNA (version 1.51) [51] was used to analyze the co-expression of mRNA and lncRNA with the criteria of weight value and R^2^ value more than 0.8. The overlapped targets of anti-sense and cis were used for further analysis. Then, KOBAS software (version: 2.1.1) [52] was used to identify the potential function of the target genes of DE lnRNAs with the criteria of *p* value less than 0.05.

### Plasmid constructions and cell transfection

According to the sequence of lncRNA *TCONS_00007391*, the RNA interference target sequence was designed as following: siRNA1: GCCTTCTGGACAGTGCCTGAA; siRNA2: GGATCCAGGAGCCTTCAACAT; siRNA3: GCTCATCTAAGAGCAACAGAA. The oligo sequence was synthesized and named as shRNA1, shRNA2, and shRNA3. After annealing, the double-stranded DNA vector (PCR product) was formed. The PLVX-EF1α-IRES-puro vector was digested with double restriction enzymes (*BamH I* and *EcoR I*) to produce the line plasmid. The obtained PCR product was then cloned into the line plasmid. The ligated products were transformed into *Escherichia coli* cells overnight culture. Positive single bacteria were sequenced. Then, the vector was transfected into chicken macrophages and the fluorescence expression was observed at 48 h. Cells from blank, shRNA-NC, shRNA1, shRNA2, and shRNA3 were harvested and used to isolate the total RNA. The RNA was reverse transcribed into cDNA, and the interference efficiency was detected by RT-qPCR. Full-length lncRNA *TCONS_00007391* was amplified by PCR and inserted into the pcDNA3.1 vector with *Hind III* and *EcoR I* restriction sites to produce pcDNA3.1-lncRNA. The primer for pcDNA3.1-lncRNA is displayed in Table S2. After macrophages were transfected with pcDNA3.1 or pcDNA3.1-lncRNA for 48 h, differently treated cells were collected to identify the overexpression efficiency with RT-qPCR.

2,000 bp of the upstream of *CD86* transcription start site was selected to construct the vector of its promoter. The *CD86* promoter fragment were amplified using 5’ unidirectional deletion specific primers containing *BamH I* and *EcoR I* restriction enzyme sites, respectively. The PCR products were cloned into pGL3-basic luciferase reporter vector (Progema, Madison, WI, USA) using T4 DNA ligase (TaKaRa, Dalian, China). After enzyme digestion and sequencing identification, the recombinant plasmids were extracted using EndoFree Mini Plasmid Kit II (Tiangen, Beijing, China), and named pGL3-CD86.

### Dual-luciferase reporter assay

When the cells reached 70–80% confluence, 1 × 10^5^ cells were seeded in 24-well plates. To verify the relationship between lncRNA *TCONS_00007391* and *CD86*, each recombinant plasmid (800 ng) was co-transfected with internal vector pRL-TK (20 ng) using Lipofectamine™ 8000 reagent according to the manufacturer’s protocol. After 48 h post-transfection, the luciferase activity was detected using the dual luciferase reporter assay system (Vazyme, Nanjing, China) and the pGL3-basic vector was used as a negative control. The firefly luciferase and Renilla luminescence activities were measured in a multi-function microplate reader (Biotek, Winooski, VT, USA).

### Verification of RNAseq data via RT-qPCR

RT-qPCR was used to determine the reliable of RNAseq data. Primer sequences of *CD86*, *TLR7*, *MAPK14*, *PRKCB*, *CD80*, *TCONS_00007391*, *ENSGALG00000049035*, *TCONS_00038895*, *ENSGALG00000037400*, and *TCONS_00007916* were displayed in Table S1. Macrophages from Control and APEC group were collected and used to extract total RNA for subsequent RT-qPCR experiment.

### RT-qPCR

RT-qPCR was conducted using a SYBR® Premix Ex Taq™ II Kit (Takara, Dalian, China). The thermal cycling conditions of RT-qPCR were as follows: denaturation for 3 min at 95 °C, 40 cycles for 10 s at 95 °C, 58 °C for 30 s, and then 72 °C for 30 s. Relative expression of the lncRNAs/genes were calculated using the 2^−∆∆Ct^ method. *GADPH* was utilized as an internal control. The formula of ΔΔCt is (Ct of lncRNA/gene in test group—Ct of *GAPDH* in test group)—(Ct of lncRNA/gene in control group—Ct of *GAPDH* in control group).

### Samples collection

The expression patterns of *TCONS_00007391* and *CD86* were investigated in chicken different tissues. Briefly, eight healthy sanhuang adult roosters with uniform body weight were purchased from Wangyuan Livestock and Poultry Breeding Co., Ltd (Shandong, China). The birds were kept under conventional housing conditions without any vaccinations. The roosters (n = 8) were euthanized by CO_2_ inhalation. A total of ten tissues was collected, including heart, liver, lung, cecum, stomach, duodenum, cerebrum, cerebellum, ileum, and spleen. Then, all the harvested tissues were stored at − 80 °C for subsequent total RNA extraction and RT-qPCR experiment.

### Cell viability assay

A density of 1 × 10^5^ cells/well was seeded in a 96-well plate containing 100 μL of medium per well. Then, cells were divided into four groups: (1) Control: cells that were neither infected APEC nor transfected with plasmids; (2) APEC: cells that were infected with APEC for 24 h; (3) oelncRNA + APEC: cells that were first transfected with overexpression of lncRNA *TCONS_00007391* vector (oelncRNA) for 48 h, and then infected with APEC for 24 h; (4) shRNA + APEC: cells that were first transfected with RNA interference vector of lncRNA *TCONS_00007391* (shRNA) for 48 h, and then infected with APEC for 24 h. The viability of chicken macrophages from different groups (Control, APEC, oelncRNA + APEC, and shRNA + APEC) was determined using Cell Counting Kit 8 (CCK8) (Vazyme, Nanjing, China). The cells were incubated for 2 h in 10 μL of CCK8 solution. The absorbance (optical density, OD) was measured at 450 nm using a microplate reader (DR-200Bs, Diatek, Wuxi, China).

### Data analysis

Statistical analysis was conducted using a one-way ANOVA and the Turkey Honestly Significant (HSD) differences test with JMP statistical software (version 15.2.1, SAS Institute). Data are expressed as the mean ± standard deviation (SD). Statistical significance was defined as* p* < 0.05.

### Supplementary Information

**Additional file 1.**

## Data Availability

The datasets presented in this study can be found in online repositories. The names of the repository/repositories and accession number(s) can be found in the article/Supplementary Material. The raw sequence reads were deposited into NCBI SRA database under accession no. PRJNA1002001.
