# Supplementary material for: Genome-wide transcriptional profiling and functional analysis of long noncoding RNAs and mRNAs in chicken macrophages associated with the infection of avian pathogenic E. coli
Source: BMC Vet Res. 2024 Feb 7;20:49. doi: 10.1186/s12917-024-03890-7 (PMC10848384; doi:10.1186/s12917-024-03890-7)
Supplement: Supplementary file 1 — Additional file 1. [file 12917_2024_3890_MOESM1_ESM.zip › supplemental files/Supplementary file 1/sh7391-2-3-sequence.docx]

TCATGAAGATTTCGATTTCTTGGCTTTATATATCTTGTGGAAAGGACGAGGATCCGGATCCAGGAGCCTTCAACATTTCAAGAGAATGTTGAAGGCTCCTGGATCCTTTTTTGAATTCTAGTTATTAATAGTAATCAATTACGGGGTCATTAGTTCATAGCCCATATATGGAGTTCCGCGTTACATAACTTACGGTAAATGGCCCGCCTGGCTGACCGCCCAACGACCCCCGCCCATTGACGTCAATAATGACGTATGTTCCCATAGTAACGCCAATAGGGACTTTCCATTGACGTCAATGGGTGGAGTATTTACGGTAAACTGCCCACTTGGCAGTACATCAAGTGTATCATATGCCAAGTACGCCCCCTATTGACGTCAATGACGGTAAATGGCCCGCCTGGCATTATGCCCAGTACATGACCTTATGGGACTTTCCTACTTGGCAGTACATCTACGTATTAGTCATCGCTATTACCATGGTGATGCGGTTTTGGCAGTACATCAATGGGCGTGGATAGCGGTTTGACTCACGGGGATTTCCAAGTCTCCACCCCATTGACGTCAATGGGAGTTTGTTTTGGCACCAAAATCAACGGGACTTTCCAAAATGTCGTAACAACTCCGCCCCATTGACGCAAATGGGCGGTAGGCGTGTACGGTGGGAGGTCTATATAAGCAGAGCTGGTTTAGTGAACCGTCAGATCCGCTAGCGCTACCGGTCGCCACCATGGCCCAGTCCAAGCACGGCCTGACCAAGGAGATGACCATGAAGTACCGCATGGAGGGCTGCGTGGACGGCCACAAGTTCGTGATCACCGGCGAGGGCATCGGCTACCCCTTCAAGGGCAAGCAGGCCATCAACCTGTGCGTGGTGGAGGGCGGCCCCTTGCCCTTCGCCGAGGACATCTTGTCCGCCGCCTTCATGTACGGCAACCGCGTGTTCACCGAGTACCCCCAGGACATCGTCGACTACTTCAAGAACTCCTGCCCCGCCGGCTACACCTTGGGACCGCTCCTTCCTGTTCGAGGACGGCGCCGTGT
