# Supplementary material for: Genome-wide transcriptional profiling and functional analysis of long noncoding RNAs and mRNAs in chicken macrophages associated with the infection of avian pathogenic E. coli
Source: BMC Vet Res. 2024 Feb 7;20:49. doi: 10.1186/s12917-024-03890-7 (PMC10848384; doi:10.1186/s12917-024-03890-7)
Supplement: Supplementary file 1 — Additional file 1. [file 12917_2024_3890_MOESM1_ESM.zip › supplemental files/Table S1.docx]

Table S1 Primers for the candidate genes or lncRNAs in RT-qPCR experiment

| Name | Forward primer (5’-3’) | Reverse primers (5’-3’) |
| --- | --- | --- |
| *GADPH* | GTCGGAGTCAACGGATTTGG | GTTCTCAGCCTTGACAGTGCC |
| *CD86* | CTTGCTACTTCCCAAACTC | TCATCCACAATCCCTACAT |
| *IL8* | GAGTTCACTGACCACCCT | TGCCTGAGCCATACCTTT |
| *TNFA* | CGTTCGGGAGTGGGCTTTA | TTGTGGGACAGGGTAGGG |
| *IL6* | GGAGAAATGCCTGACGAA | GGATTGTGCCCGAACTAA |
| *IL1β* | CGCTTCATCTTCTACCGC | CCAGTCACAATAAATACCTCCA |
| *TLR7* | GGCTGTGAATGAATGGGTG | GCTGAATGCTCTGGGAAAG |
| *MAPK14* | ATTGTGAAATGCCAGAAAC | GTCATCTCATCATCCGTGT |
| *PRKCB* | CAGCAGGTTGGGAGGTTTA | CCAGCTTCAGGTCTCGGTA |
| *CD80* | TGCTTATTGCGTTGACCCT | ATTCCAGCGAACCCTTTGT |
| *TCONS_00007391* | GACCCACATAATGGTTGAG | AAGGTCCCACAGAGTTTCC |
| *ENSGALG00000049035* | TTTTGTCTCCGTTCCTACC | TCAATCACCATTCCAGCAT |
| *TCONS_00038895* | GCCCTTCACTTCATTACTT | ACTTTCCAAGCTGTGACCC |
| *ENSGALG00000037400* | TGGGTTTGTACGGGTCAGC | TGTGGAGCAGGGCAGCAGAG |
| *TCONS_00007916* | TCATCACATCAAATACCCACT | GCTCCCTCAGAAGACAGAA |
