# Supplementary material for: Genome-wide transcriptional profiling and functional analysis of long noncoding RNAs and mRNAs in chicken macrophages associated with the infection of avian pathogenic E. coli
Source: BMC Vet Res. 2024 Feb 7;20:49. doi: 10.1186/s12917-024-03890-7 (PMC10848384; doi:10.1186/s12917-024-03890-7)
Supplement: Supplementary file 1 — Additional file 1. [file 12917_2024_3890_MOESM1_ESM.zip › supplemental files/Table S2.docx]

Table S2 The primer for overexpression of TCONS_00007391 vector

| Name | Forward primer (5’-3’) | Reverse primers (5’-3’) |
| --- | --- | --- |
| homologous recombination | CTGGCTAGCGTTTAAACTTAAGTACTTGTTCGTGTCCTG | ACTGTGCTGGATATCTGCAGTGGGTTAGTTTTGAAGCTG |
| T4 ligation | CCCAAGCTTAGTACTTGTTCGTGTCCTG | GGAATTCTGGGTTAGTTTTGAAGCTG |
