# Supplementary figures and images for: Genome-wide transcriptional profiling and functional analysis of long noncoding RNAs and mRNAs in chicken macrophages associated with the infection of avian pathogenic E. coli
Source: BMC Vet Res. 2024 Feb 7;20:49. doi: 10.1186/s12917-024-03890-7 (PMC10848384; doi:10.1186/s12917-024-03890-7)

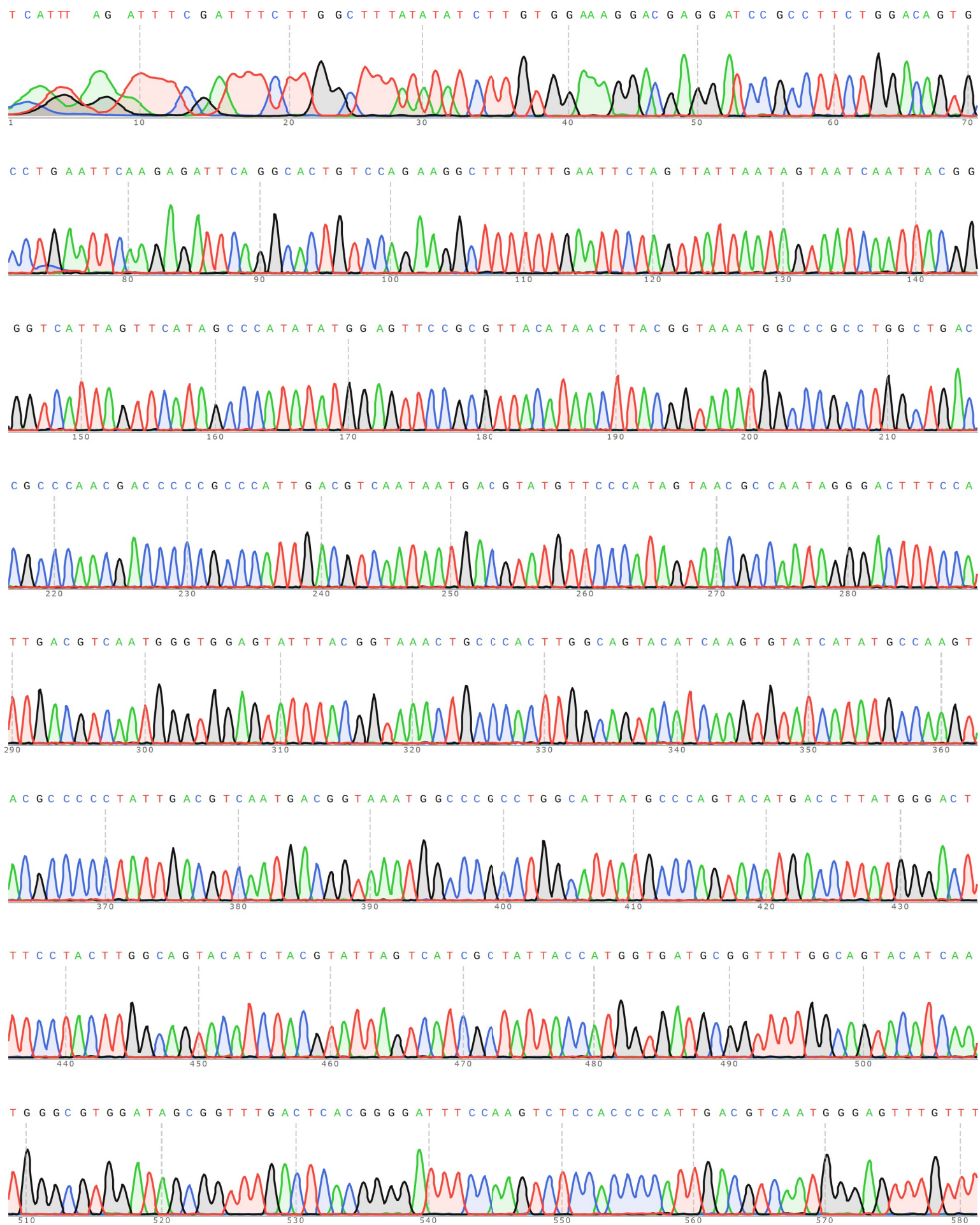

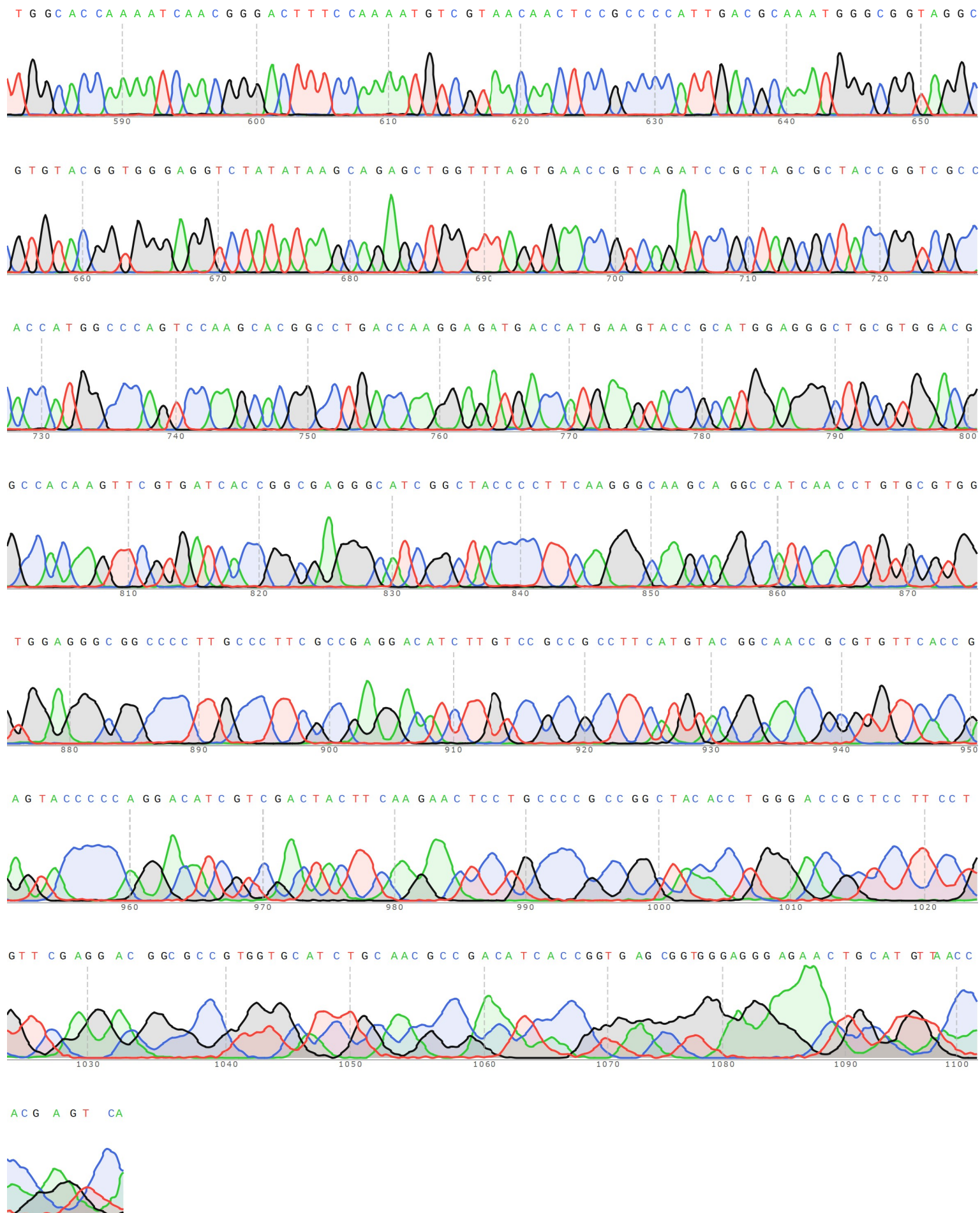

Supplement: Supplementary file 1 — Additional file 1. [file 12917_2024_3890_MOESM1_ESM.zip › supplemental files/Supplementary file 1/sh7391-1-12-peak.pdf]

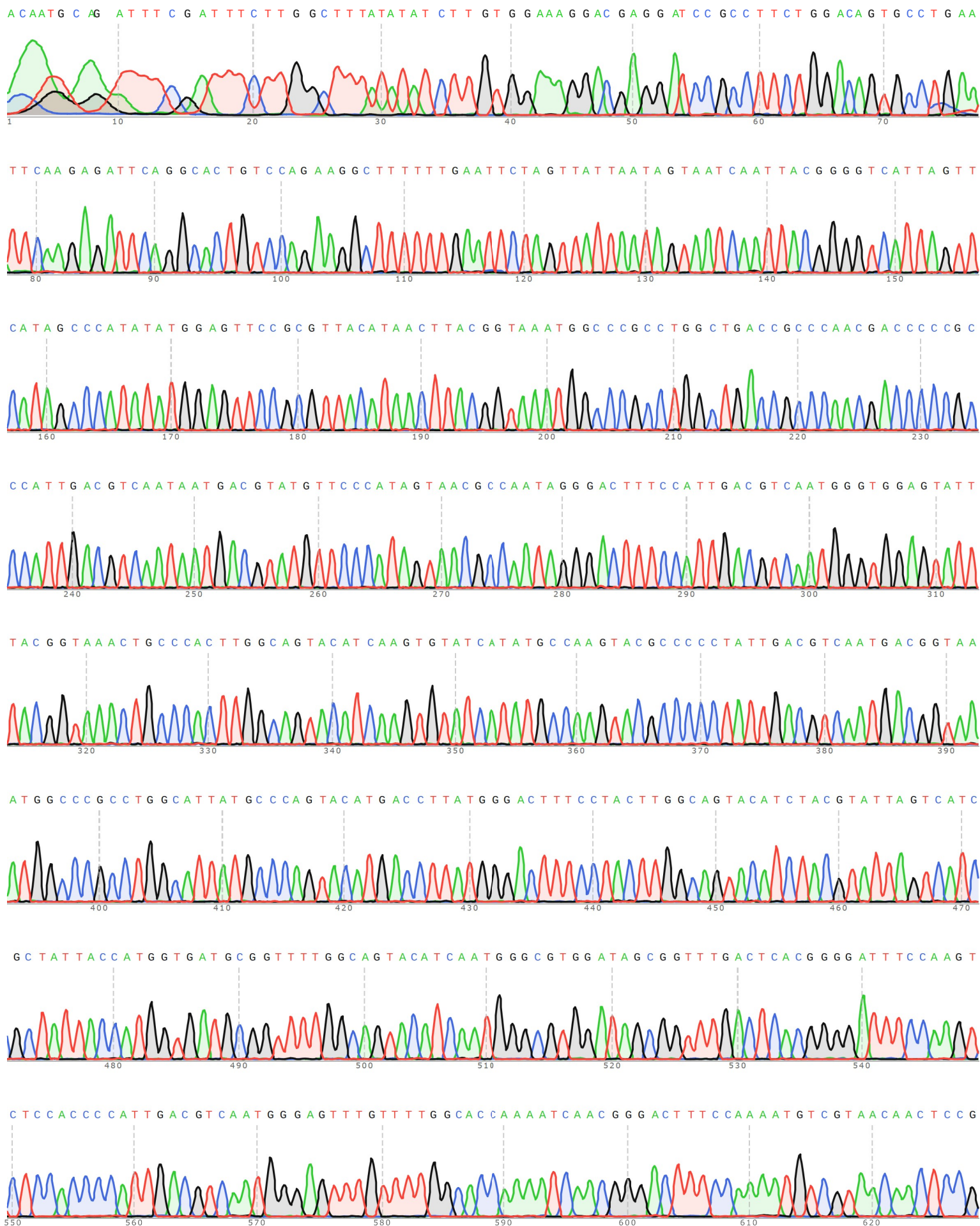

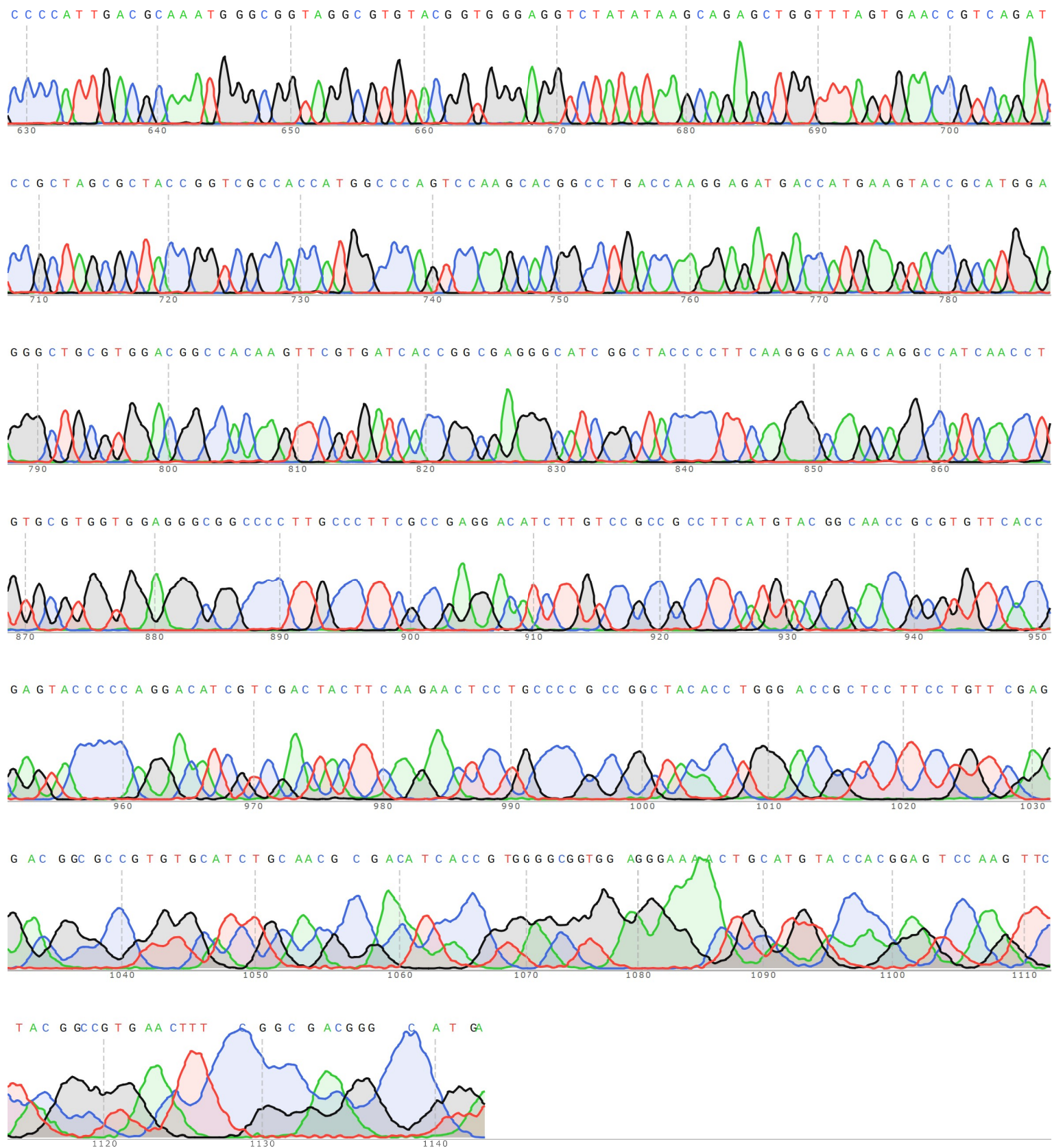

Supplement: Supplementary file 1 — Additional file 1. [file 12917_2024_3890_MOESM1_ESM.zip › supplemental files/Supplementary file 1/sh7391-1-6-peak.pdf]

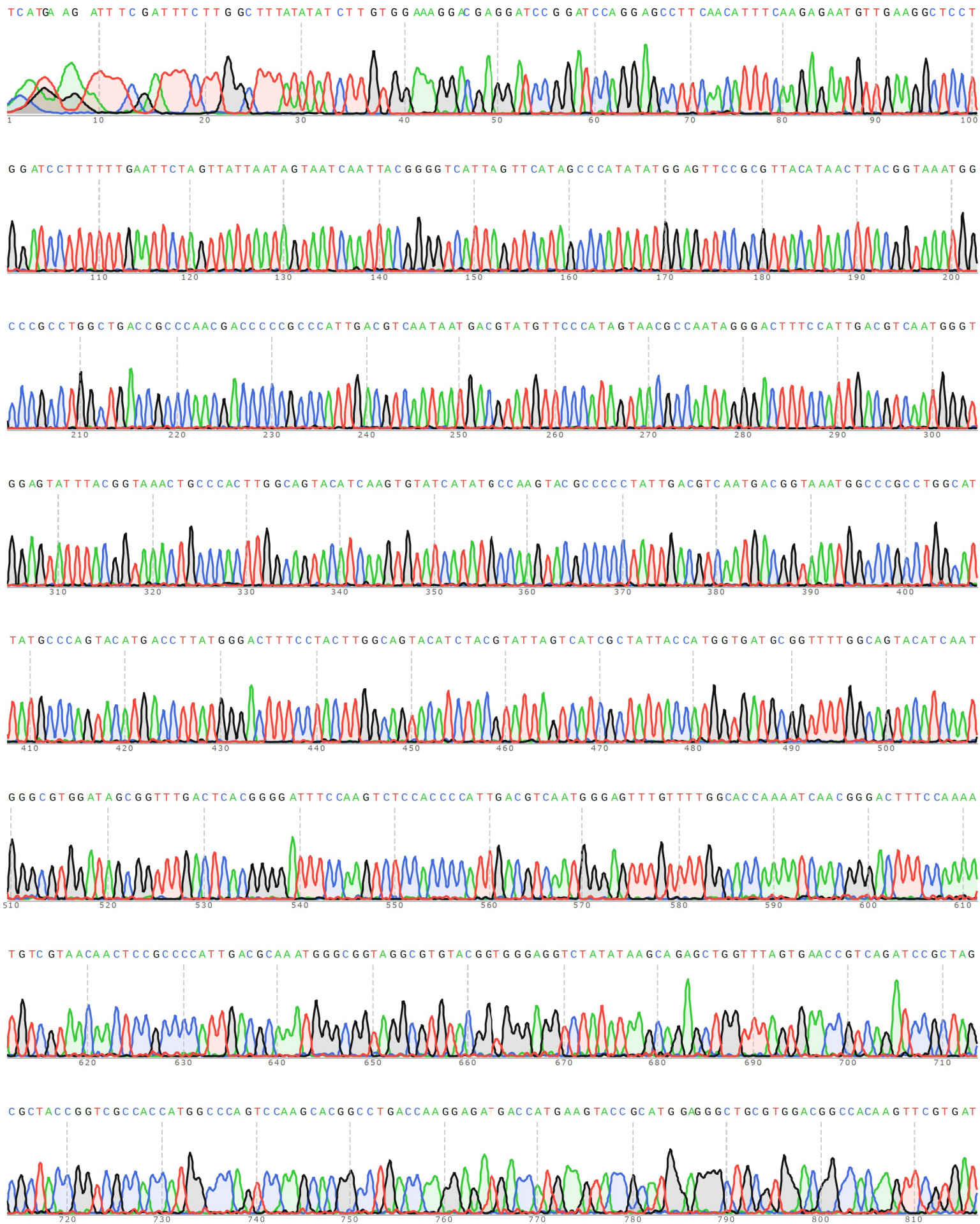

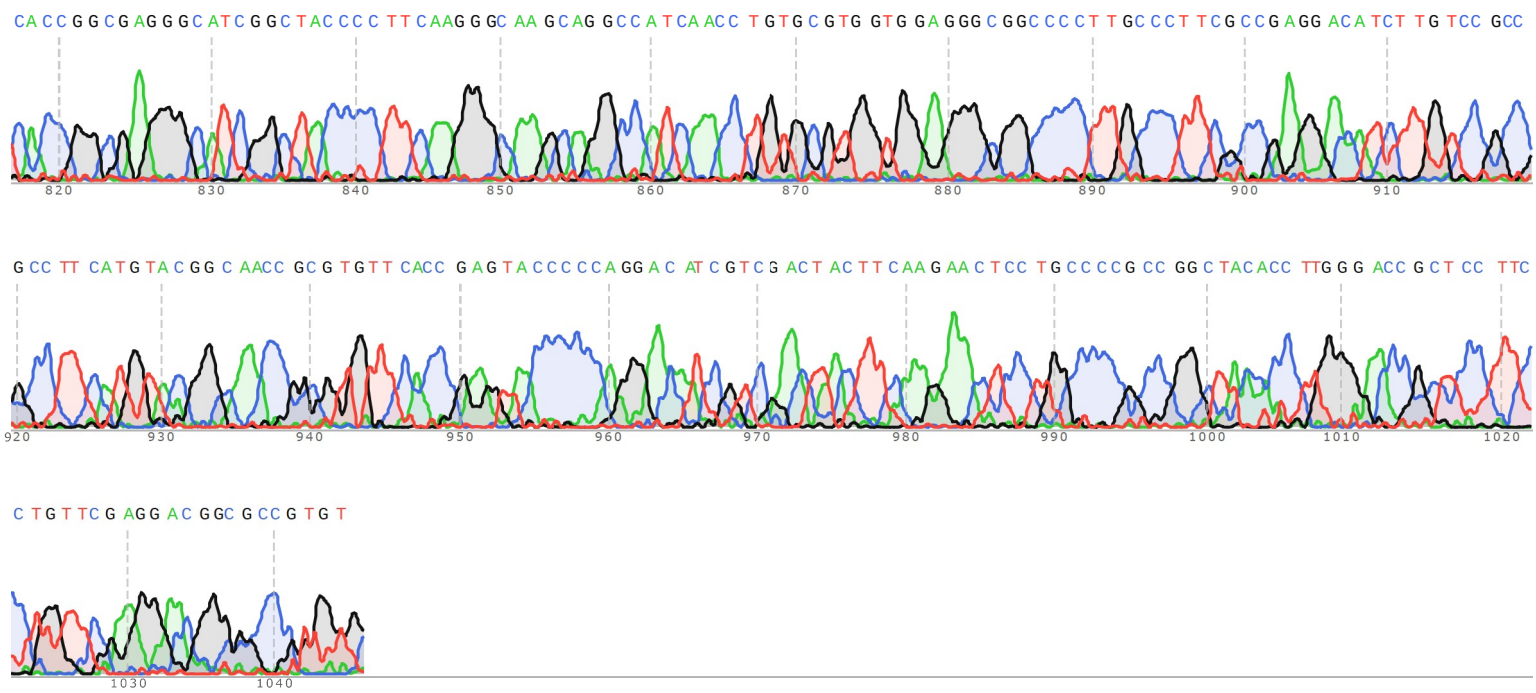

Supplement: Supplementary file 1 — Additional file 1. [file 12917_2024_3890_MOESM1_ESM.zip › supplemental files/Supplementary file 1/sh7391-2-3-peak.pdf]

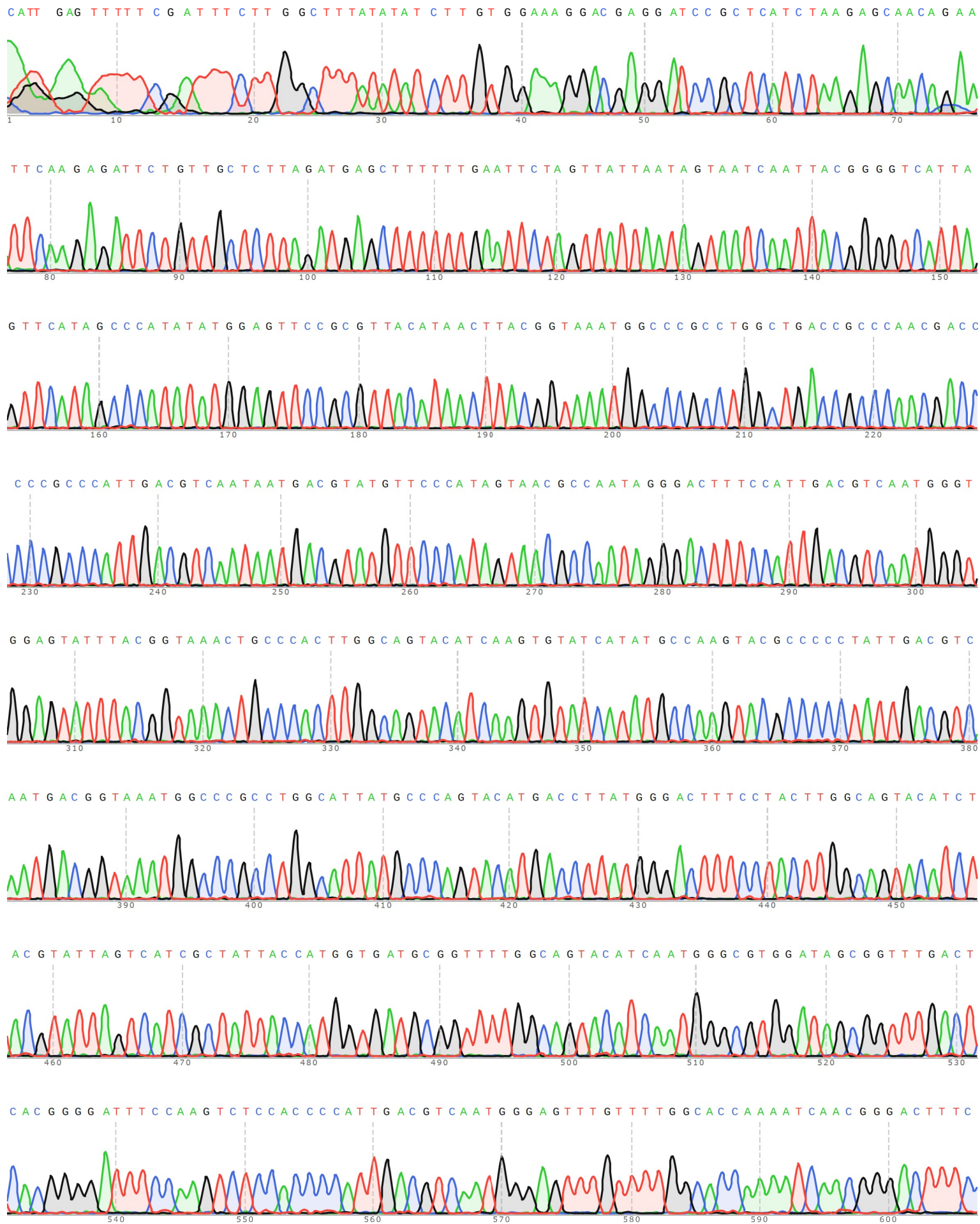

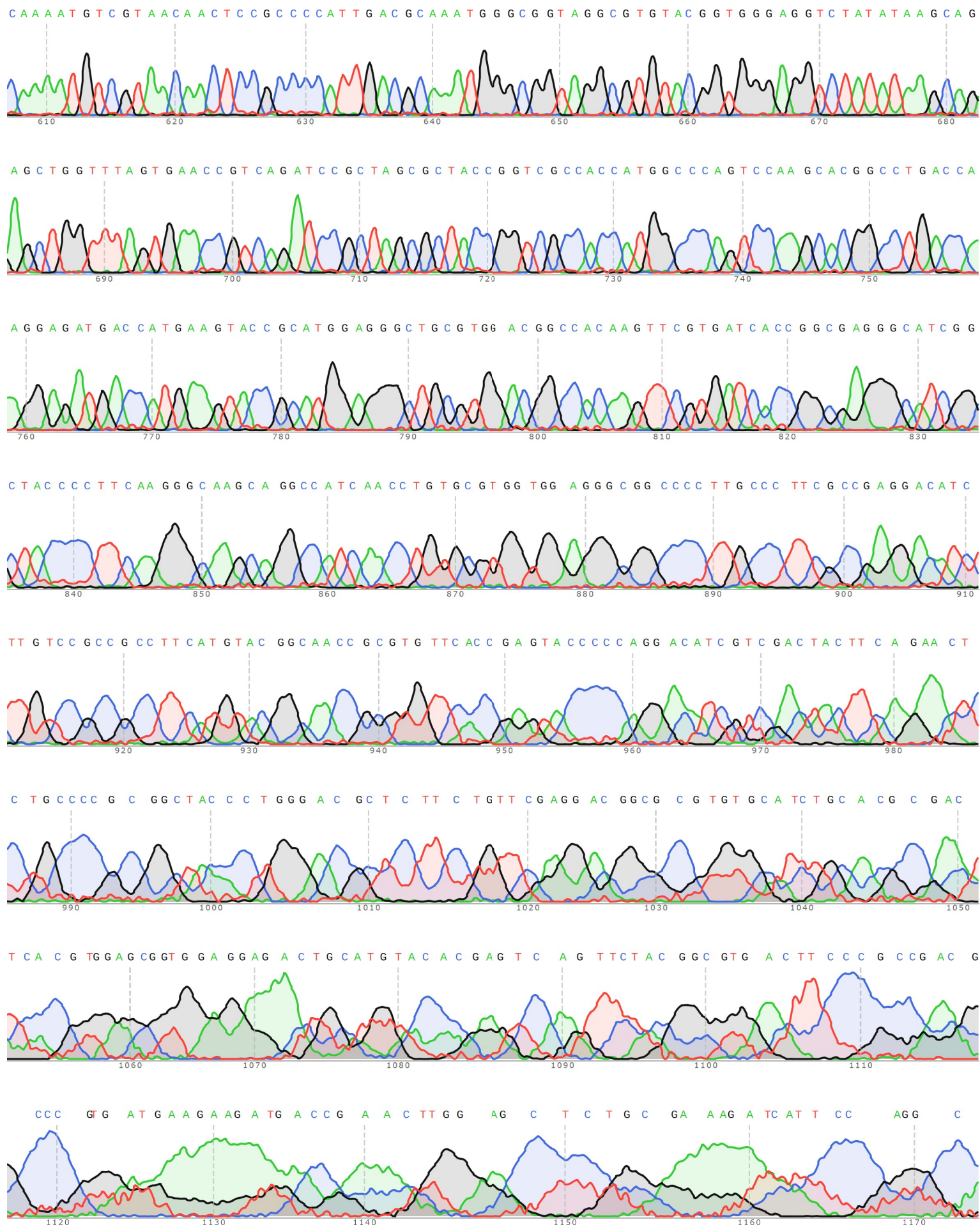

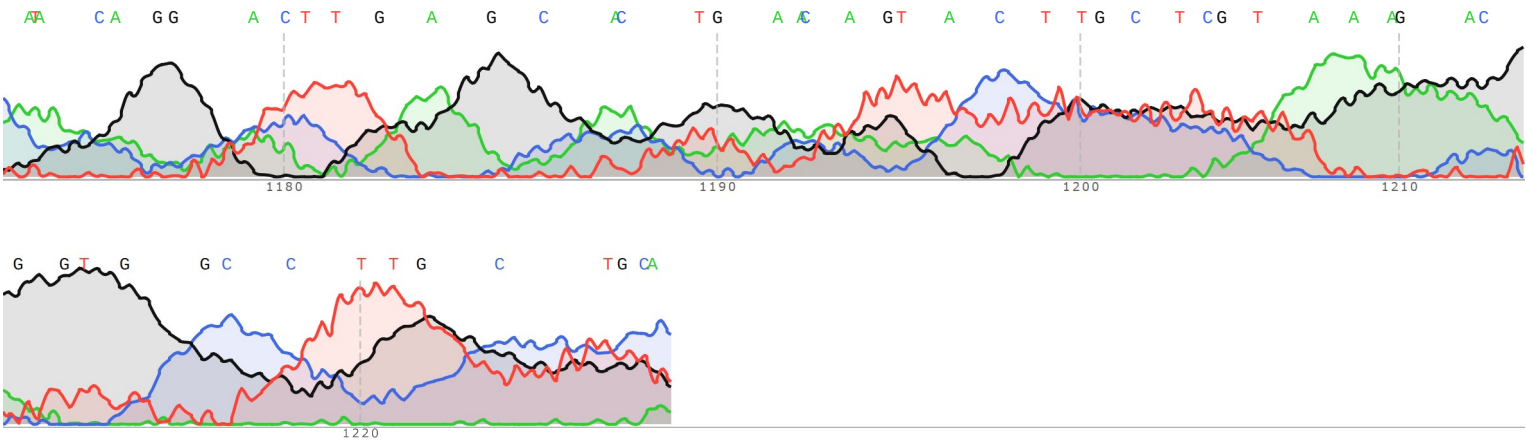

Supplement: Supplementary file 1 — Additional file 1. [file 12917_2024_3890_MOESM1_ESM.zip › supplemental files/Supplementary file 1/sh7391-3-8-peak.pdf]

Original gel

Figure 8A


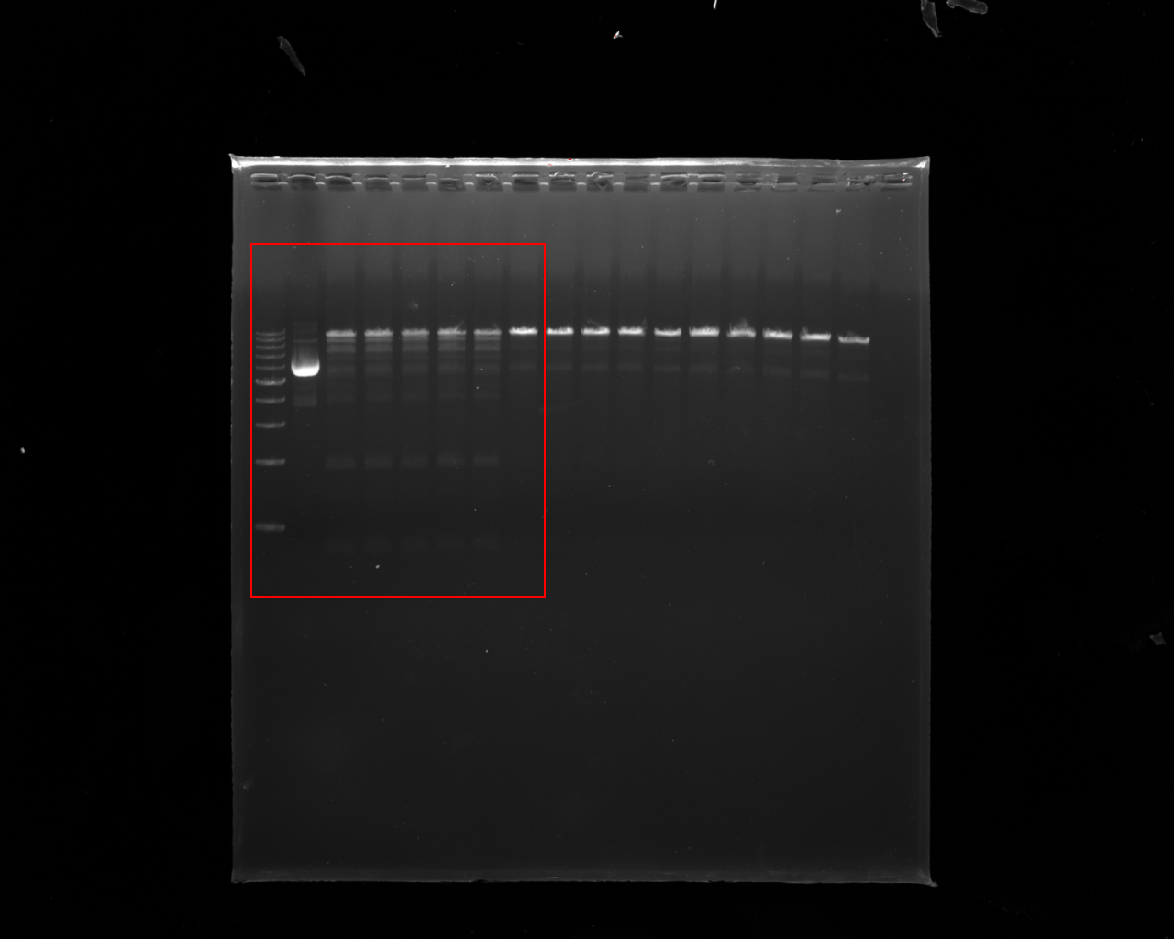

Supplement: Supplementary file 1 — Additional file 1. [file 12917_2024_3890_MOESM1_ESM.zip › supplemental files/Supplementary file 2/Original gel_Figure 8A.docx]

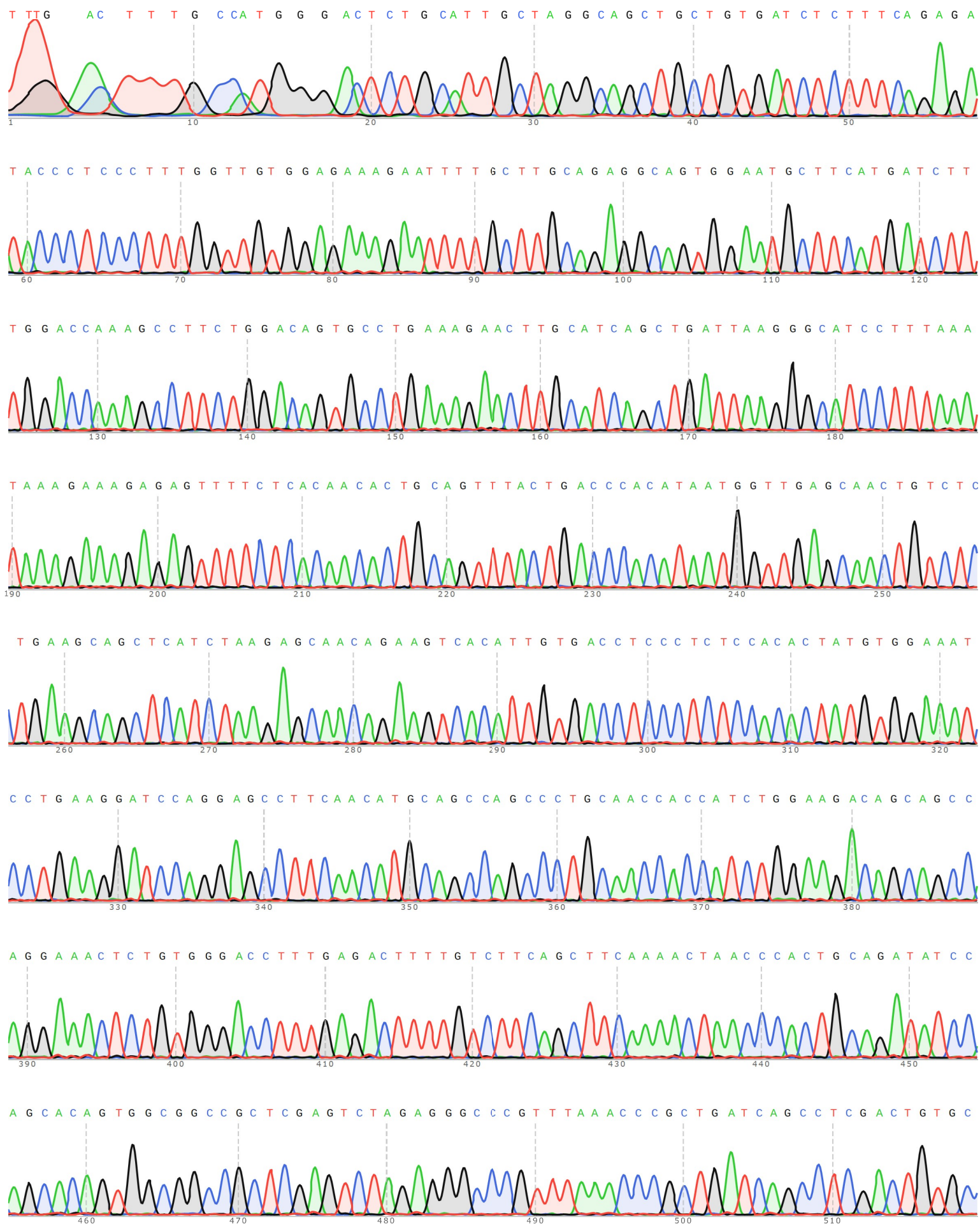

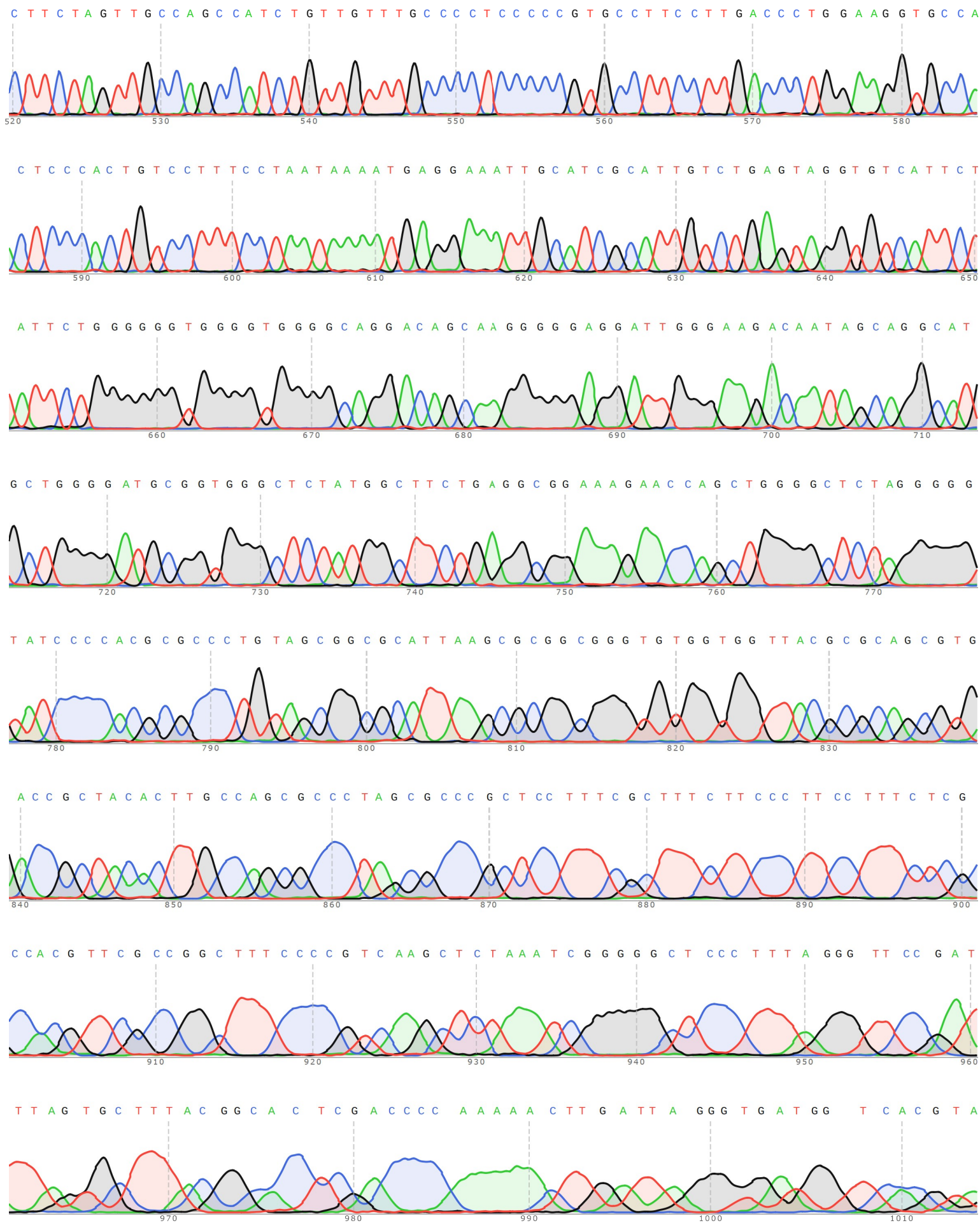

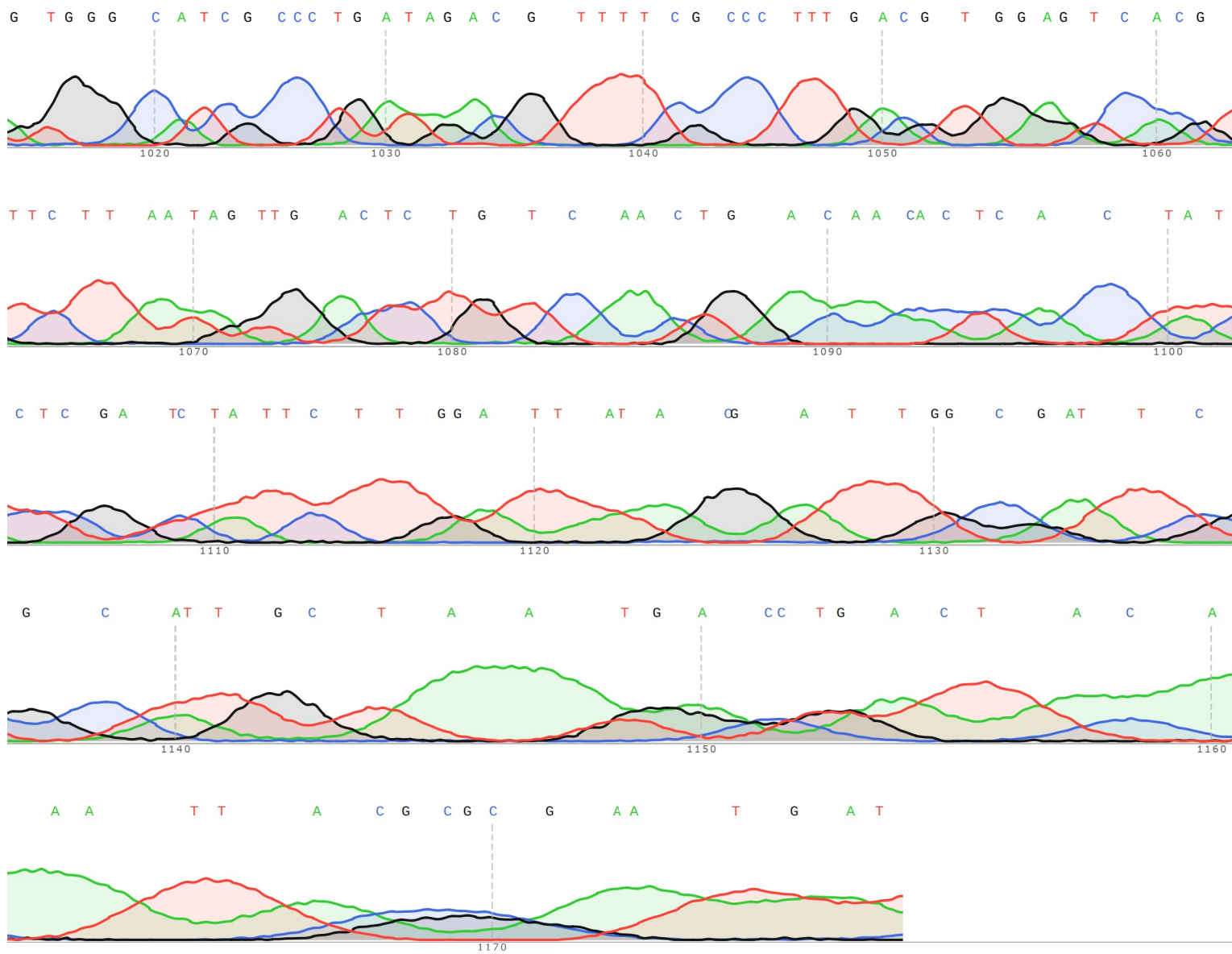

Supplement: Supplementary file 1 — Additional file 1. [file 12917_2024_3890_MOESM1_ESM.zip › supplemental files/Supplementary file 3/7391-1-peak.pdf]

Original gel

Figure 8D


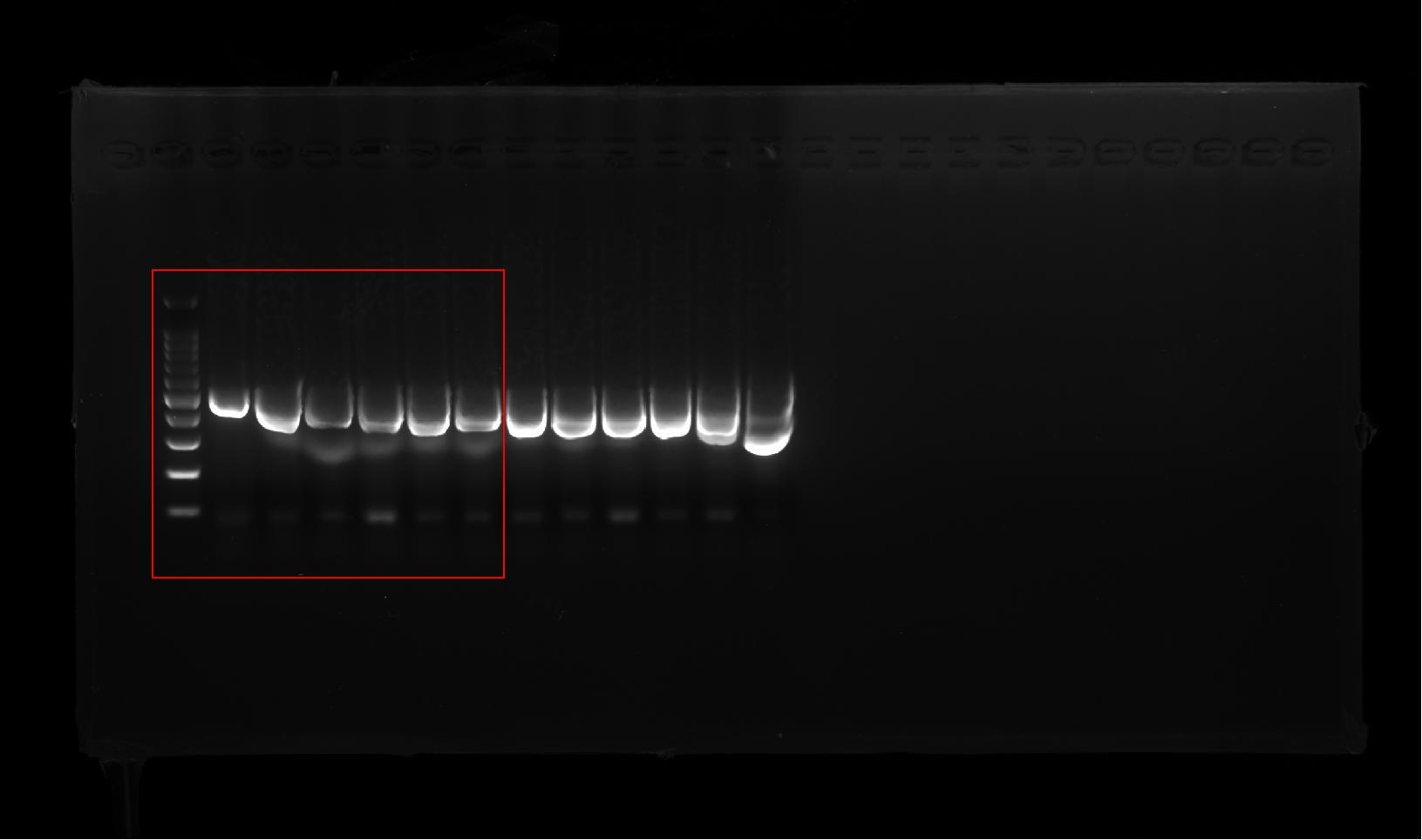

Supplement: Supplementary file 1 — Additional file 1. [file 12917_2024_3890_MOESM1_ESM.zip › supplemental files/Supplementary file 4/Original gel_Figure 8D.docx]
